# Assessing the Effect of Smokeless Tobacco Consumption on Oral Microbiome in Healthy and Oral Cancer Patients

**DOI:** 10.3389/fcimb.2022.841465

**Published:** 2022-03-31

**Authors:** Rituja Saxena, Vishnu Prasoodanan P K, Sonia Vidushi Gupta, Sudheer Gupta, Prashant Waiker, Atul Samaiya, Ashok K. Sharma, Vineet K. Sharma

**Affiliations:** ^1^ MetaBioSys Group, Department of Biological Sciences, Indian Institute of Science Education and Research Bhopal, Bhopal, India; ^2^ Department of Surgical Oncology, Bansal Hospital, Bhopal, India; ^3^ Department of Gastroenterology, Inflammatory Bowel & Immunology Research Institute, Cedars Sinai Medical Center, Los Angeles, CA, United States

**Keywords:** oral microbiome shift, tobacco consumption, oral squamos cell carcinoma, dignostic biomarker, microbiome & dysbiosis

## Abstract

Oral cancer is a globally widespread cancer that features among the three most prevalent cancers in India. The risk of oral cancer is elevated by factors such as tobacco consumption, betel-quid chewing, excessive alcohol consumption, unhygienic oral condition, sustained viral infections, and also due to dysbiosis in microbiome composition of the oral cavity. Here, we performed an oral microbiome study of healthy and oral cancer patients to decipher the microbial dysbiosis due to the consumption of smokeless-tobacco-based products and also revealed the tobacco-associated microbiome. The analysis of 196 oral microbiome samples from three different oral sites of 32 healthy and 34 oral squamous cell carcinoma (OSCC) patients indicated health status, site of sampling, and smokeless tobacco consumption as significant covariates associated with oral microbiome composition. Significant similarity in oral microbiome composition of smokeless-tobacco-consuming healthy samples and OSCC samples inferred the possible role of smokeless tobacco consumption in increasing inflammation-associated species in oral microbiome. Significantly higher abundance of *Streptococcus* was found to adequately discriminate smokeless-tobacco-non-consuming healthy samples from smokeless-tobacco-consuming healthy samples and contralateral healthy site of OSCC samples from the tumor site of OSCC samples. Comparative analysis of oral microbiome from another OSCC cohort also confirmed *Streptococcus* as a potential marker for healthy oral microbiome. Gram-negative microbial genera such as *Prevotella*, *Capnocytophaga*, and *Fusobacterium* were found to be differentially abundant in OSCC-associated microbiomes and can be considered as potential microbiome marker genera for oral cancer. Association with lipopolysaccharide (LPS) biosynthesis pathway further confirms the differential abundance of Gram-negative marker genera in OSCC microbiomes.

## Introduction

Oral cancer is among the 10 most prevalent cancers globally and ranks among the three most common cancers in India (Reichart, 2001; Sharma et al., 2018). The high incidence of oral cancer could be attributed to a combined effect of specific risk factors such as tobacco consumption, exposure to carcinogenic agents, and insufficient access to newly developed diagnostic aids that delays the diagnosis of oral cancer (Sharma et al., 2018). The low-income groups in India are the most affected by oral cancer due to high consumption of smokeless tobacco products such as gutkha and pan masala, whose main ingredients are tobacco, areca nut, and betel quid (Nair et al., 2004). Habitual chewers consume tobacco with or without betel quid. In the recent decades, the availability of inexpensive and attractive sachets of betel quid substitutes has been increased in India (Sahitha, 2014). The product is essentially a flavored and sweetened dry mixture of areca nut, slaked lime, and catechu with tobacco (gutkha) or without tobacco (pan masala). Some previous studies in India that looked at the correlation between tobacco consumption and oral cancer found that 49% of the oral cancer types were attributable to tobacco chewing (Balaram et al., 2002). Tobacco chewing and poor hygiene were attributed to 95% of oral cancers in women (Muwonge et al., 2008). However, these studies only explored the association of cancer incidence with tobacco consumption and did not examine the role of dysbiosis in oral microbiome due to tobacco consumption in the progression of the disease.

Alterations or dysbiosis in human microbiome have been associated with different types of cancers and other metabolic and physiological diseases, such as colorectal cancer, diabetes, obesity, and autism (Graessler et al., 2012; Everard and Cani, 2013; Maji et al., 2018; Pulikkan et al., 2018; Saxena et al., 2018). Previous studies have made efforts to characterize the oral microbiome in several populations (Kilian et al., 2016; Acharya et al., 2017; Sarkar et al., 2017; Xian et al., 2018; Chattopadhyay et al., 2019; Burcham et al., 2020; Rai et al., 2021). In the last 5 years, multiple studies have also shown a significant variation in the microbial community between patients suffering from oral squamous cell carcinoma (OSCC) and healthy individuals (He et al., 2015; Sarkar et al., 2017; Liu et al., 2018; Perera et al., 2018; Yang et al., 2018). The studies have directly examined malignant tissue by swab or biopsy and demonstrated that the carcinogenic sites are enriched for Gram-negative Fusobacteria and Bacteroidetes, showing lower abundance of *Streptococcus* and *Rothia* spp. (Schmidt et al., 2014; Al-Hebshi et al., 2017). An earlier study examined the microflora-based differences in 45 OSCC subjects and 229 OSCC-free (control) individuals and observed that high salivary counts of *Prevotella melaninogenica*, *Capnocytophaga gingivalis*, and *Streptococcus mitis* could act as diagnostic indicators of OSCC (Mager et al., 2005).

Inflammation is shown to be one of the most critical factors among the different mechanisms and causes known for cancer induction. At the clinical level, the association between inflammation and induction of cancer has been well established in the case of oral cavity, ovaries, prostate, bladder, liver, pancreas, colon, stomach, and other sites (Rosin et al., 1994; Ness and Cottreau, 1999; Itzkowitz and Yio, 2004; Rogers and Fox, 2004; Whitcomb, 2004; Zavros et al., 2004; Palapattu et al., 2005; Hooper et al., 2009; Sharma et al., 2020). Notably, 15%–20% of the tumors are reported to be caused by microbe-induced inflammation (Tlaskalová-Hogenová et al., 2011).

Long-term exposure to tobacco and tobacco-containing products has been known to be a significant cause for OSCC worldwide (Jiang et al., 2019). The major ingredients and carcinogens found in tobacco-based products such as gutkha and pan masala are tobacco-specific nitrosamines (TSNAs) including NNK [4‐(methylnitrosamino)‐1‐(3‐pyridyl)‐1‐butanone], NNN (N‐nitrosonornicotine), and MNPN [3‐(methylnitrosamino) propionitrile], and ROS (reactive oxygen species, O–·, H_2_O_2_, OH) (Nair et al., 2004)· Chewing of tobacco with betel quid results in high exposure to carcinogenic TSNAs and has also been associated with dysbiosis in the oral microflora of tobacco-consuming individuals compared to non-consuming individuals (Reichart, 2001; Hooper et al., 2009; Sahitha, 2014). Oral conditions like leukoplakia, erythroplakia, and oral submucous fibrosis are the primary conditions of oral cancer in betel nut/tobacco chewers. The oral microbiome has been identified to play an important role in the development of oral submucous fibrosis. Long-term use of betel nut, particularly tannic acid, has shown to inhibit the growth of commensal bacteria. Cigarette smoking has also shown to alter the abundance of common taxa in the oral microbiome (Lin et al., 2014; Wu et al., 2016).

A few oral microbes are also known to produce carcinogens/procarcinogens from tobacco-based products such as nitrosamine by *Candida* and acetaldehyde (procarcinogen) by *Candida*, *Neisseria*, and *Streptococcus* (Hooper et al., 2007; Hooper et al., 2009). The reaction of nitrite with alkaloids results in nitrosamines, where nitrate-reducing bacteria play a significant role by providing nitrite for conversion (Fisher et al., 2012). Taken together, it is apparent that there is a need to further explore the impact of tobacco consumption on the oral microbiome and its role in oral cancer through conversion of tobacco-based metabolites into carcinogens. In addition, the identification of metabolites produced in the oral cavity of both healthy, tobacco-consuming, and oral cancer patients also needs to be examined (Ursell et al., 2014; Wang et al., 2014). A few salivary biomarkers such as propionylcholine and acetylphenylalanine have been proposed to be used as a biomarker for the early detection and diagnosis of OSCC (Wang et al., 2014).

Although there has been a recent surge in global studies on oral microbiome, its dysbiosis, and its association with oral cancer and gut microbiome (Ahn et al., 2012; Shillitoe, 2018), only a limited number of studies have been reported from the developing countries (Acharya et al., 2017; Batool et al., 2020; Nolan-Kenney et al., 2020; Pandey et al., 2020; Rai et al., 2021). Importantly, it is much needed to decipher the dysbiosis in oral microbiome due to the consumption of smokeless-tobacco-based products and their association with oral cancer. With this key objective, we carried out the analysis of 196 oral microbiome samples from three different oral sites of 32 healthy and 34 oral squamous cell carcinoma (OSCC) patients from Bhopal that has the highest incidence of reported oral cancer cases in India. The results obtained in this study helped to reveal the smokeless-tobacco-associated microbial community in healthy and oral cancer patients.

## Methods

### Ethics Approval and Consent to Participate

The study was reviewed and approved by the Institute Ethics Committee (IEC) of Indian Institute of Science Education and Research (IISER) Bhopal, India. The recruitment of volunteers, sample collection, and other study-related procedures were performed by following the approved guidelines and protocols, and a written-informed consent was obtained from all the volunteers prior to any study-related procedures.

### Subject Recruitment and Swab Sample Collection

The study cohort comprised of 66 subjects, which included 34 OSCC patients and 32 healthy individuals. The OSCC patients were recruited with the association of Navodaya Cancer Hospital and Bansal Hospital in Bhopal, India. Experienced oncologists at the hospitals carried out diagnosis of all the cases through biopsy and other standard diagnostic methods. Patients with only primary untreated OSCC (higher than stage III) who have not undergone treatments (i.e., radiotherapy/chemotherapy, surgery) were recruited as described in an earlier study (Sami et al., 2020). The healthy individuals did not have any history of OSCC and did not show the presence of any mucosal lesions. Individuals with any history of diabetes mellitus or immune-system-related diseases were excluded from the study. All the healthy subjects did not undergo any antibiotic treatment for 1 month prior to the sample collection. Other information about the individuals such as gender, age, and tobacco-consuming habits were recorded before the sample collection (**Supplementary Table S1**).

**Figure 1:**
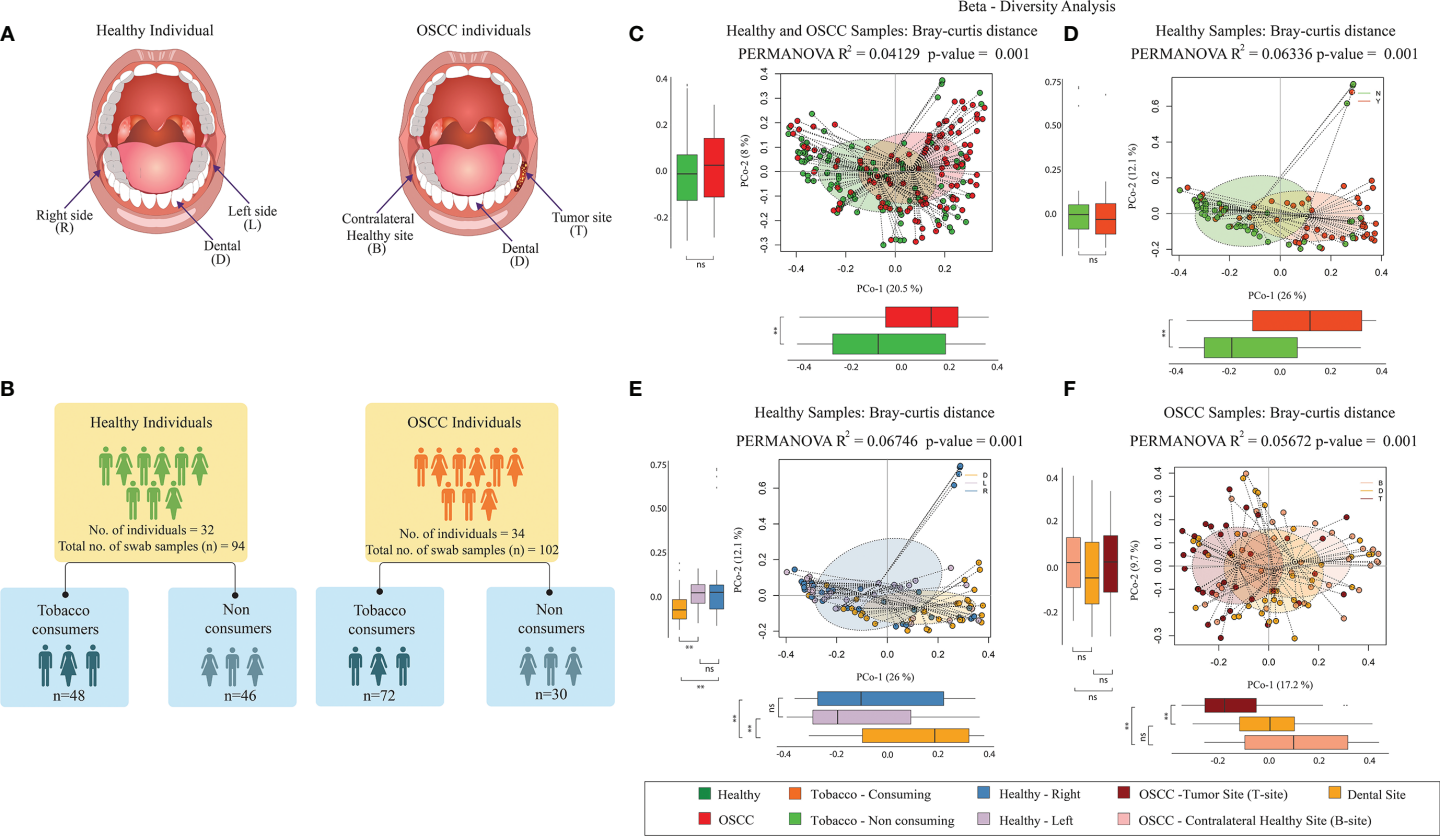
Microbiome diversity in Healthy and OSCC samples. **(A)** Sampling sites of oral cavity considered in this study. For the healthy individuals, swab samples were collected from the right (abbreviated as “R”) and left (abbreviated as “L”) buccal site and the dental plaque or biofilm (abbreviated as “D”). For the OSCC patients, the swab samples were collected from the cancer lesion site or tumor site (abbreviated as “T”), its anatomically matched contralateral normal buccal site (abbreviated as “B”), and dental plaque or biofilm (abbreviated as “D”). **(B)** Number of samples used in this study. The healthy group consisted of 48 smokeless tobacco-consuming (TC-H) and 46 non-consuming (NTC-H) samples. The OSCC group consists of 72 smokeless-tobacco-consuming (TC-OSCC) and 30 non-consuming (NTC-OSCC) samples. **(C)** Principal coordinate analysis considering intersample Bray–Curtis distance between all 196 samples. Samples were tagged based on the health status. **(D)** Principal coordinate analysis considering intersample Bray–Curtis distance between 94 healthy samples. Samples were tagged based on the smokeless tobacco consumption status. **(E)** Principal coordinate analysis considering intersample Bray–Curtis distance between 94 healthy samples. Samples were tagged as left buccal site, right buccal site, and dental site. **(F)** Principal coordinate analysis considering intersample Bray–Curtis distance between 102 OSCC samples. Samples were tagged as tumor site, contralateral healthy site, and dental site. **p < 0.01; ns, not significant.

Swab samples were collected from three oral sites of both patients and healthy individuals, as described in an earlier study (Schmidt et al., 2014). A total of 198 swab samples were collected, 102 from the OSCC patients and 96 from the healthy individuals (**Figure 1A**). For the patients, the swab samples were collected from the cancer lesion site or tumor site (abbreviated as “T”), its anatomically matched contralateral normal buccal site (abbreviated as “B”), and dental plaque or biofilm (abbreviated as “D”). For the healthy individuals, swab samples were collected from the right (abbreviated as “R”) and left (abbreviated as “L”) buccal site and the dental plaque or biofilm (abbreviated as “D”). To collect samples from the tumor site, the lesion was dried by blotting with gauze and was stroked with a sterile cotton swab (Hi-Media Laboratories Ltd., Mumbai, India). The swab was stroked across the lesion 10 times, applying gentle downward pressure. It was then rotated to 180°, and the other side of the swab was stroked 10 times across the lesion in the same manner. Anatomically matched contralateral normal, dental, and buccal sites were sampled in the same manner. The swabs were placed into the collection tube, snap frozen immediately after collection, and were stored at −80°C until DNA extraction.

### Metagenomic DNA Extraction From Swab Samples

Metagenomic DNA was extracted from the swab samples using DNeasy blood and tissue kit (Qiagen, MD, USA) as per the manufacturer’s instructions with minor modifications. The head from the swabs was cut and placed in 2-ml Eppendorf tubes containing 750 µl phosphate-buffered saline (PBS). The swabs were then vortexed at full speed for 2–3 min and removed/discarded after squeezing on the sides of the tube to recover maximum cells/sample. After this, 2 µl of lysozyme (15 mg/ml), 180 µl ATL buffer, and 20 µl Proteinase K was added to the tube and incubated at 56°C for 10 min after a brief vortexing. A total of 200 µl of 100% ice-chilled ethanol was then added to the sample and vortexed briefly. The remaining steps were performed as per the manufacturer’s instructions, and the extracted DNA was stored at −20°C. The DNA concentration was measured using Qubit ds DNA HS kit on a Qubit 2.0 fluorometer (Life Technologies, Carlsbad, CA, USA).

### Sample Collection and Metagenomic DNA Extraction of Commercial Tobacco Products

The tobacco-product samples considered in this study for microbiome analysis were obtained from various commercially available tobacco products. Three samples each of commercially available tobacco sachets (T-GU, T-BL, and T-V1), cigarettes (CG-GF, CG-WNC, and CG-GL), and beedi (BD-SH, BD-M55, and BD-50) were collected to examine the abundance of microbial species. For metagenomic DNA extraction, 200 mg of tobacco from each commercial product was mixed with 10 µl lysozyme and 1 ml PBS in lysing matrix E tubes (MP Biomedicals LLC, CA, USA). The tubes were incubated at 37°C for 30 min, after which 10 µl of proteinase K (20 mg/ml) and 50 µl of 10% sodium dodecyl sulfate (SDS) was added to the tube and vortexed briefly. The tubes were then incubated at 55°C for 30 min, and then, bead beating was performed for 2 min at 4,800 rpm using a bead beater. The samples were then centrifuged at 10,000×*g* for 5 min, and the supernatant was collected in a 2-ml microcentrifuge tube. This step was repeated, and the supernatant collected was mixed with 500 µl of 100% ethanol. The solution was passed through a column (Qiagen Inc., Hilden, Germany), to which 500 µl of AW1 from DNeasy blood and tissue kit (Qiagen Inc., Hilden, Germany) was added, and the column was centrifuged at ≥6,000×*g* for 1 min. The flow-through was discarded, and 500 µl of AW2 was added and centrifuged at 14,000 rpm for 3 min. The flow-through was then discarded, and the column was again centrifuged at 14,000 rpm for 1 min to remove excess buffer. Elution buffer (10 mM tris at pH 8.5) was used to elute the DNA from the column. The DNA concentration was measured using Qubit ds DNA HS kit on a Qubit 2.0 fluorometer (Life Technologies, Carlsbad, CA, USA), and the extracted DNA was stored at −20°C until further procedures.

### 16S rRNA Gene Amplification and Sequencing

Equal concentration of metagenomic DNA (~5 ng) was used for PCR amplification of bacterial 16S rRNA gene V3 hypervariable region. The amplification was performed using Illumina Nextera XT adapter-ligated eubacterial V3 region-specific primers, 341F and 534R, with five different base modifications (Wang and Qian, 2009; Soergel et al., 2012). Nucleotide bases were incorporated in different numbers between the primer and adapter sequence to increase the overall sequence diversity of the samples, thereby improving the quality of the sequenced data. Bacterial DNA samples were divided into six groups and amplified using the six different primers. Primer sequences for amplification of bacterial 16S rRNA gene V3 region are as below (the base inclusions are marked in bold).

The underlined regions in all the primer sequences are the Illumina Nextera XT adapter overhangs, whereas the non-underlined regions are the primer sequences known to target V3 region of eubacterial 16S rRNA gene.

1. 341F-ADA
  5′ TCGTCGGCAGCGTCAGATGTGTATAAGAGACAGCCTACGGGAGGCAGCAG 3′
  534R-ADA
  5′ GTCTCGTGGGCTCGGAGATGTGTATAAGAGACAGATTACCGCGGCTGCTGGC 3′
2. 341F_ADA_1B
  5′ TCGTCGGCAGCGTCAGATGTGTATAAGAGACAG **T**CCTACGGGAGGCAGCAG 3′
  534R_ADA_1B
  5′ GTCTCGTGGGCTCGGAGATGTGTATAAGAGACAG **C**ATTACCGCGGCTGCTGGC 3′
3. 341F_ADA_2B
  5′ TCGTCGGCAGCGTCAGATGTGTATAAGAGACAG **CT**CCTACGGGAGGCAGCAG 3′
  534R_ADA_2B
  5′ GTCTCGTGGGCTCGGAGATGTGTATAAGAGACAG **CT**ATTACCGCGGCTGCTGGC 3′
4. 341F_ADA_3B
  5′ TCGTCGGCAGCGTCAGATGTGTATAAGAGACAG **CAT**CCTACGGGAGGCAGCAG 3′
  534R_ADA_3B
  5′ GTCTCGTGGGCTCGGAGATGTGTATAAGAGACAG **ACT**ATTACCGCGGCTGCTGGC 3′
5. 341F_ADA_4B
  5′ TCGTCGGCAGCGTCAGATGTGTATAAGAGACAG **TCAT**CCTACGGGAGGCAGCAG 3′
  534R_ADA_4B
  5′ GTCTCGTGGGCTCGGAGATGTGTATAAGAGACAG **CTAT**ATTACCGCGGCTGCTGGC 3′
6. 341F_ADA_5B
  5′ TCGTCGGCAGCGTCAGATGTGTATAAGAGACAG **CTACT**CCTACGGGAGGCAGCAG 3′
  534R_ADA_5B
  5′ GTCTCGTGGGCTCGGAGATGTGTATAAGAGACAG **CATCT**ATTACCGCGGCTGCTGGC 3′

The optimized PCR conditions included the following: initial denaturation at 94°C for 5 min, followed by 35 cycles of denaturation at 94°C for 30 s, annealing at 69°C for 30 s, extension at 72°C for 30 s, and a final extension cycle at 72°C for 5 min. Paq5000 DNA polymerase (Agilent Technologies, Santa Clara, CA, USA) was used for amplification from swab samples, and 5% dimethyl sulfoxide (DMSO) was added to the reaction to improve the concentration of the amplified products from the metagenomic template.

After evaluating the amplified products on 2% w/v agarose gel, the products were purified using Ampure XP kit (Beckman Coulter, Brea, CA, USA). Amplicon libraries were prepared by following the Illumina 16S metagenomic library preparation guide. The libraries were evaluated on 2100 Bioanalyzer using DNA1000 kit (Agilent Technologies, Santa Clara, CA, USA) to estimate the library size. The libraries were further quantified on a Qubit 2.0 fluorometer using Qubit dsDNA HS kit (Life Technologies, USA) and by quantitative PCR (qPCR) using KAPA SYBR FAST qPCR Master mix and Illumina standards and primer premix (KAPA Biosystems, Wilmington, MA, USA), following the Illumina-suggested protocol. Libraries in equal concentrations were loaded on Illumina NextSeq 500 platform using NextSeq 500/550 v2 sequencing reagent kit (Illumina Inc., USA), and 150 bp paired-end sequencing was performed at the Next-Generation Sequencing (NGS) Facility, IISER Bhopal, India. Since we did not obtain successful PCR amplification in two samples, they were excluded out of the 198 samples. Therefore, a total of 196 swab samples (102 from OSCC patients and 94 from healthy individuals) were considered for further analysis.

### 16S rRNA Amplicon Analysis

The raw sequence data were subjected to ambiguity filtering and quality filtration using NGSQC toolkit (Patel and Jain, 2012), and the paired-end reads were assembled using FLASH (Magoč and Salzberg, 2011). A total of 74,255,974 (median = 759,600) high-quality V3 amplicon reads per sample were used for the analyses. Operational taxonomic unit (OTU) picking was carried out using QIIME v1.9 at 97% identity against the Greengenes database (v13_5, https://greengenes.secondgenome.com/). The reads that failed to cluster in closed reference OTU picking were clustered using *de novo* OTU picking. The representative sequences obtained from *de novo* OTU picking were aligned against the Greengenes database using BLAT, and the taxonomic assignment was performed using Lowest Common Ancestor (LCA) algorithm (Kent, 2002; DeSantis et al., 2006; Caporaso et al., 2010). SILVA database was also used to further validate the taxonomic assignment of the OTUs. The OTU count per sample was normalized by dividing it with total number of reads in the corresponding sample. The number of reads assigned to different taxonomic classes (mainly phylum, genus, and species) was calculated, and the taxonomic composition was evaluated for each sample. PICRUSt algorithm was employed to predict the bacterial functions in the healthy and OSCC groups (Langille et al., 2013).

### Statistical Analyses of Amplicon Data

All statistical analyses were performed using R software. The α-diversity metrics (observed species, Shannon, Simpson, and Chao1) and β-diversity (unweighted UniFrac distance, weighted UniFrac distance, and Bray–Curtis distance) were calculated using QIIME on rarefied OTU counts at equal depths. The genus abundance tables were analyzed for the identification of discriminating genera using LEfSe (Segata et al., 2011) and Boruta (Kursa et al., 2010).

### Saliva Collection, Preparation, and UPLC-MS

Sixteen saliva samples were collected for the metabolomic analysis using ultraperformance liquid chromatography–mass spectrometry (UPLC-MS), comprising of 11 samples from healthy individuals and five samples from OSCC patients (**Supplementary Table S1**). All the volunteers refrained from consuming tobacco, drinking, eating, or follow any oral hygiene procedures for at least 1 h prior to the sample collection and were asked to rinse their mouth with clean water. The saliva samples were collected between 9–11 a.m. by following the previously described protocol (Wang et al., 2014). Approximately, 3 ml of unstimulated whole saliva was collected from all the volunteers and was transported to the laboratory in liquid nitrogen. The samples were then centrifuged at 12,000 rpm for 20 min at 4°C to remove food remnants, insoluble materials, and cell debris. Supernatant was aliquoted to fresh tubes in equal amounts (400 µl) and frozen at −80°C until further procedures. For the metabolite extraction, the frozen saliva was thawed at room temperature. A mixture of acetonitrile/methanol (75:25 v/v, 800 µl) was added to the saliva (400 µl) in a 1.5-ml Eppendorf tube to precipitate proteins. After vortexing for 60 s, the mixture was incubated for 10 min at room temperature, and the samples were centrifuged at 12,000 rpm for 20 min at 4°C. The supernatant was filtered through syringe filters (0.22 µm) before the UPLC-MS analysis. LC-MS separation was performed on Bruker microTOF QII high-resolution mass spectrometer coupled to Waters Acquity UPLC system, on an C18 column (50 mm × 2.1 mm i.d., 1.7 mm, Waters, Milford, USA) at the central mass facility, IISER Bhopal.

### UPLC-MS Data Pre-treatment and Analysis

UPLC-MS was used for the identification of metabolites from the oral cavity of healthy individuals and OSCC patients. MZMine2 (Pluskal et al., 2010) was used for the downstream analysis of the raw data. Peak detection was carried out by generation of mass lists (detected ions) for each scan followed by detection of chromatograms using the Chromatogram builder. After this, smoothing and separation of individual peaks in the chromatograms were performed using the Chromatogram deconvolution. The normalized peak intensities were used for further downstream analysis. Peak identification was carried out by searching the peaks against MetaCyc and KEGG metabolome databases using online database search in MZMine2. Normalized peak intensities for each m/z/RT group from all the samples were used for further analysis along with the metadata information.

### Statistical Analysis of Metabolomic Data

Calculating cumulative abundances of peaks belonging to the same metabolites led to the identification of total 336 metabolites. To decipher the metabolic patterns, principal coordinate analysis was carried out based on Bray–Curtis distance matrices calculated using vegan and ape package in R. Significantly discriminating metabolites between healthy and OSCC patients were identified using random forest machine learning algorithm. The importance of each metabolite for their classification ability were accessed from the mean decrease in accuracy values. Selected top 30 metabolites (MDA>1) were used for building the heatmap in R. Association study between oral metabolome and microbiota was carried out with the abundance of microbial genera in each sample and their metabolomic profiles using “CCrepe” package in R.

## Results

The cohort constructed in this study comprised of 66 subjects including 34 oral squamous cell carcinoma (OSCC) patients and 32 healthy individuals. We collected a total of 196 swab samples from three different oral sites of healthy and OSCC individuals. The healthy group consisted of 16 smokeless-tobacco-consuming (TC-H) and 16 non-consuming individuals (NTC-H) with age between 21 and 60 years (32.15 ± 9.19, mean ± Stdev), whereas 24 OSCC patients were smokeless tobacco consumers (TC-OSCC), and 10 were non-consumers (NTC-OSCC) with age ranged between 23 and 75 years (48.61 ± 12.76, mean ± Stdev) (**Figures 1A, B**; **Supplementary Table S1**). The V3 hypervariable region of bacterial 16S rRNA gene was sequenced and analyzed to compare the variations between the different groups including smokeless-tobacco-consuming and non-consuming healthy samples and OSCC samples.

### Effect of Health Status, Smokeless Tobacco Consumption, and Sampling Sites on Oral Microbiome Composition

Different indexes (Shannon, Simpson, and Chao 1) were employed to estimate the α-diversity of the bacterial community in different groups of samples (**Supplementary Figure S1**). Alpha diversity analysis using Chao1 index revealed a significantly lower (*p*-value =3.02E−06) microbial diversity in the OSCC patients than healthy individuals (**Supplementary Figure S1A**). Smokeless-tobacco-consuming healthy samples were observed to have a higher intra-sample diversity (Shannon and Simpson index) than smokeless-tobacco-non-consuming samples (**Supplementary Figure S1B**). By contrast, smokeless-tobacco-non-consuming OSCC samples were observed to have a higher intra-sample diversity compared to smokeless-tobacco-consuming OSCC samples (**Supplementary Figure S1C**). Analysis focused on the sampling sites revealed that samples from dental plaque had higher intra-sample diversity (Shannon and Simpson index) than left and right buccal sites in healthy samples. Higher bacterial diversity is observed in healthy dental plaque compared to diseased samples (**Supplementary Figure S1D**).

Principal coordinate analysis (PCoA) based on Bray–Curtis inter-sample distance (PERMANOVA R^2^ = 0.0413, *p*-value = 0.001) and unweighted UniFrac – intersample distance (PERMANOVA, R^2^ = 0.0454, p-value = 0.001) matrices showed clear separation between healthy and OSCC samples (**Figure 1C**; **Supplementary Figure S2A**). Principle coordinate1 explained 20.5% variation in the data (Bray–Curtis distance-based analysis), and it significantly separated healthy and OSCC samples. Similarly, PCoA based on unweighted UniFrac distance revealed the significant separation (both PCo-1 and PCo-2) between healthy and OSCC samples (**Supplementary Figure S2A**). In Bray-Curtis distance-based PCoA of smokeless-tobacco-consuming and non-consuming healthy samples, PCo-1 explained 26% variation among healthy samples and significantly separated the two groups (PERMANOVA R^2^ = 0.0634, *p*-value = 0.001, **Figure 1D**). Similarly, unweighted UniFrac distance-based PCo revealed a significant separation between smokeless-tobacco- and non-consuming samples across PCo-2 (PERMANOVA, R^2^ = 0.03099, *p*-value = 0.001, **Supplementary Figure S2B**). No significant clustering was observed between smokeless-tobacco-consuming and non-consuming OSCC samples (**Supplementary Figures S2D, E**). Based on the sampling site of healthy subjects, dental samples showed separate cluster in both Bray–Curtis (PERMANOVA, R^2^ = 0.0674, p-value = 0.001, **Figure 1E**) and unweighted UniFrac distance (PERMANOVA, R^2^ = 0.0388, p-value = 0.001, **Supplementary Figure S2C**) analysis. Samples from left and right buccal site did not show any significant difference in inter-sample diversity (**Supplementary Figure 2C**). By contrast, OSCC samples showed clustering based on sampling sites. Samples from tumor site showed separate cluster in both Bray–Curtis (PERMANOVA, R^2^ = 0.0567, *p*-value = 0.001, **Figure 1F**) and unweighted UniFrac distance (PERMANOVA, R^2^ = 0.0374, *p*-value = 0.001, **Supplementary Figure S2F**) analysis.

To further assess the effect of various covariates on microbiome profiles, PERMANOVA was performed using Bray–Curtis distances (**Supplementary Table S3**). The Bray–Curtis distances showed significant association with health status, smokeless tobacco consumption, and site of sampling and does not show significant association with other covariates such as age, gender, smoking, and alcohol consumption (*p* > 0.001). Healthy samples (n=94, number of individuals = 32) showed significant association with smokeless tobacco consumption (p-value = 0.0002). These observations indicate smokeless tobacco consumption as one of the drivers of dysbiosis in healthy oral microbiome. It is also apparent that the oral health status has a substantial role in explaining inter-sample variation in oral microbiome. Notably, the oral microbiome composition in oral cancer patients was not driven by smokeless tobacco consumption status, whereas its variation was observed according to the sample site (tumor and contralateral healthy site).

### Similarity in Oral Microbiome Composition of OSCC and Healthy Smokeless-Tobacco-Consuming Samples

**Figure 2:**
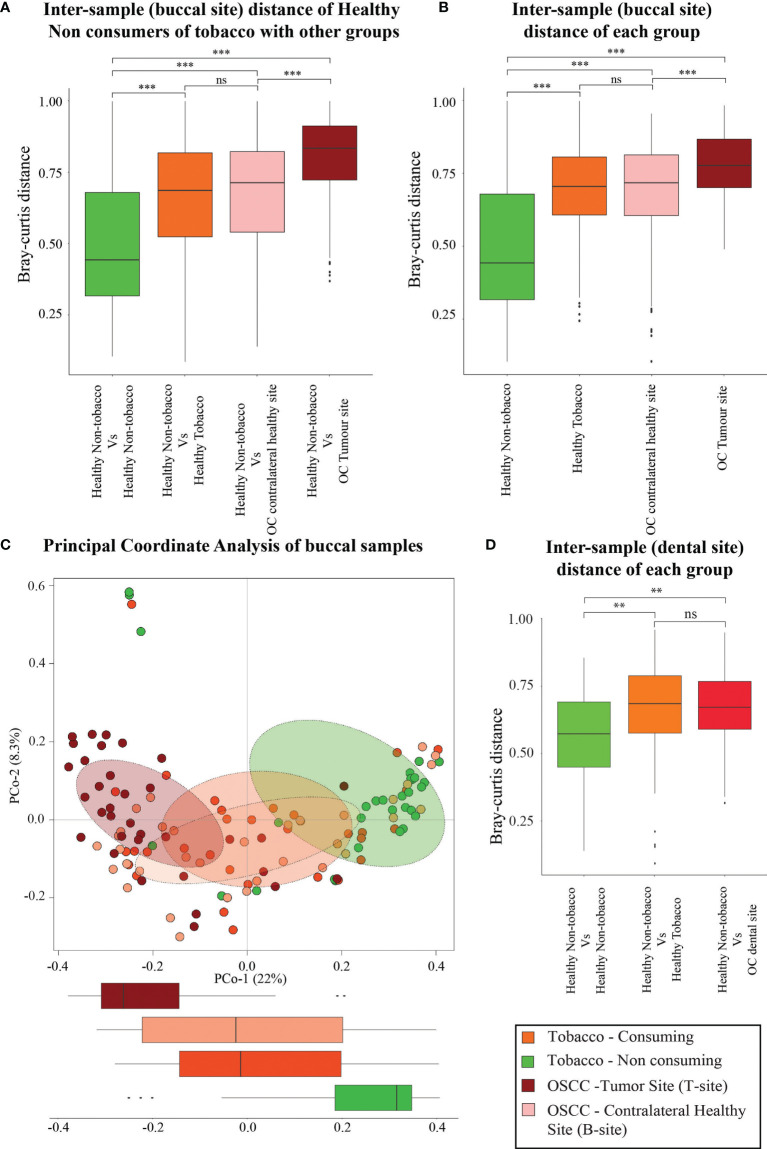
Analysis based on average intersample Bray–Curtis distance of samples from buccal and dental sites. **(A)** Comparison between intersample Bray–Curtis distance of oral microbiome (buccal site) of healthy smokeless tobacco non-consumers from healthy smokeless tobacco consumers, tumor site (T-site) of OSCC patients, and contralateral healthy site (B-site) of OSCC patients. **(B)** Intersample Bray–Curtis distance of oral microbiome from buccal site of healthy smokeless tobacco non-consumers, healthy smokeless tobacco consumers, tumor site (T-site) of OSCC patients, and contralateral healthy site (B-site) of OSCC patients. **(C)** Principal coordinate analysis considering intersample Bray–Curtis distance between buccal samples of healthy smokeless tobacco non-consumers, healthy smokeless tobacco-consumers, tumor site (T-site) of OSCC patients, and contralateral healthy site (B-site) of OSCC patients. **(D)** Comparison between intersample Bray–Curtis distance of dental microbiome of healthy smokeless tobacco non-consumers from healthy smokeless tobacco consumers and OSCC patients. **p < 0.01; ***p < 0.001; ns, not significant.

Since the microbial population structure in soft and hard tissues show substantial differences, we analyzed the microbiome composition in soft tissues (buccal sites) separately. Analysis based on average inter-sample Bray–Curtis distance of buccal samples from healthy smokeless tobacco non-consumers with other groups showed that microbiome composition in htumor site was significantly different from healthy buccal sites of smokeless tobacco non-consumers (**Figure 2A**). Interestingly, the average inter-sample distance between healthy smokeless-tobacco consumers and non-consumers, and healthy smokeless tobacco non-consumers and OSCC (contralateral to tumor site) patients were similar (**Figure 2A**). Average inter-sample Bray–Curtis distance in each group was also evaluated (**Figure 2B**), and microbiome in OSCC tumor site showed higher inter-sample distance compared to all other groups (Kruskal–Wallis test, *p* < 0.01). In addition, no significant difference in average Bray–Curtis inter-sample distance was observed between samples from healthy smokeless tobacco consumers and OSCC buccal (contralateral healthy) sites. Principal Coordinate Analysis of buccal samples of these four groups indicated a clear separation between samples from tumor site of OSCC patients and healthy smokeless tobacco non-consumers (**Figure 2C**). Interestingly, higher overlap was observed between samples from contralateral to tumor site and healthy smokeless tobacco consumers (**Figure 2C**). Similarly, dental samples also showed a comparable average inter-sample distance in healthy smokeless tobacco consumers and OSCC patients (**Figure 2D**). These observations indicate a probable deterministic shift of healthy oral microbiome to a more dispersed microbial community composition in smokeless-tobacco-consuming individuals that showed similarity with the community composition in OSCC buccal site.

### Differential Abundance of *Bacteroidetes* and *Firmicutes* in Smokeless-Tobacco-Consuming and Non-consuming Healthy Samples

**Figure 3:**
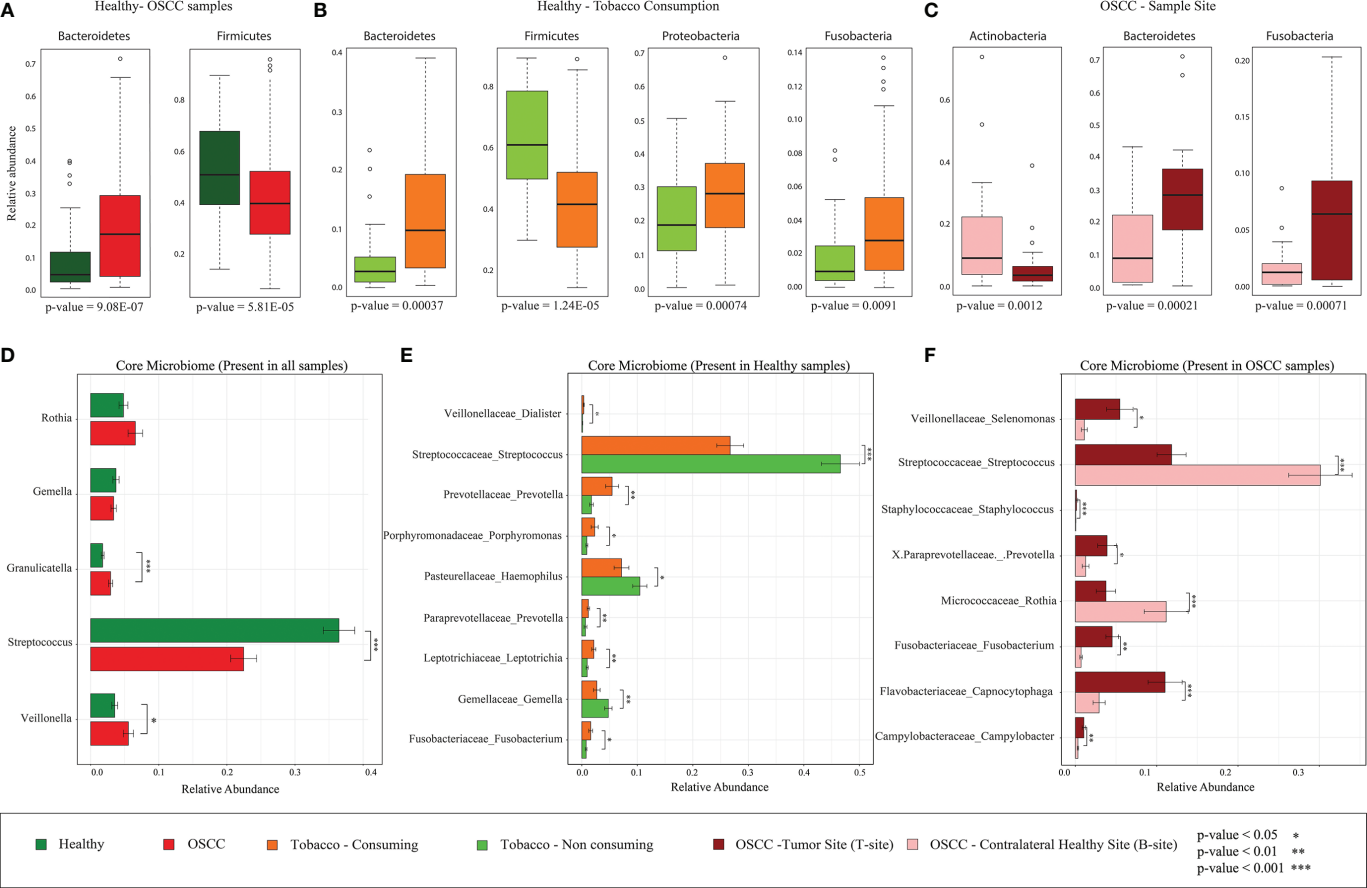
Differentially abundant bacterial phyla and core microbiome (genus level) of healthy and OSCC samples. **(A)** Differentially abundant bacterial phyla in healthy and OSCC samples identified using Boruta. All 196 samples were considered for this analysis. **(B)** Differentially abundant bacterial phyla in smokeless-tobacco-consuming and non-consuming healthy samples identified using Boruta. All 94 healthy samples were considered for this analysis. **(C)** Differentially abundant bacterial phyla in the tumor site and contralateral healthy site of OSCC samples identified using Boruta. All 102 OSCC samples were considered for this analysis. **(D)** Relative abundance of core genera with >1% abundance in healthy and OSCC samples (n=196). The significance levels were indicated based on Wilcoxon rank-sum test. **(E)** Relative abundance of core genera with >1% abundance in healthy smokeless-tobacco-consuming and non-consuming samples (n=94). The significance levels were indicated based on Wilcoxon rank-sum test. **(F)** Relative abundance of core genera with >1% abundance in the tumor site and contralateral healthy site of OSCC samples (n=102). The significance levels were indicated based on Wilcoxon rank-sum test.

Several previous oral microbiome studies have concluded that a healthy oral cavity harbors a plethora of microorganisms broadly belonging to six significant phyla, *namely*, *Firmicutes*, *Bacteroidetes*, *Proteobacteria*, *Actinobacteria*, *Spirochaetes*, and *Fusobacteria.* The presence of these phyla in Indian oral microbiome reaffirms the presence of core phyla in oral microbiome (**Supplementary Figure S3A**). Significantly discriminating phyla between the healthy and OSCC samples were identified using Boruta and LEfSe (Kursa et al., 2010) (see *Methods*). The analysis revealed that phylum *Firmicutes*, the highest abundant phylum in oral microbiome, is significantly higher in the healthy samples than in OSCC samples (FDR-corrected *p* = 0.00006, **Figure 3A**). Boruta and LEfSe also identified *Fusobacteria* and *Proteobacteria* with a similar trend but were not significant based on Wilcoxon rank-sum test, whereas *Bacteroides* showed a significantly lower abundance in the healthy group (FDR-corrected *p* = 9.077e−07, **Figure 3A**). *Bacteroidetes* and *Firmicutes* showed a similar trend in the healthy oral samples based on smokeless tobacco consumption status. *Bacteroides* (FDR-corrected *p* = 0.0004), *Proteobacteria* (FDR-corrected *p* = 0.0007), and *Fusobacteria* (FDR-corrected p-value = 0.0090) are differentially abundant in smokeless-tobacco-consuming healthy samples, and *Firmicutes* (FDR-corrected *p* = 1.24E-05) are differentially abundant in non-consuming healthy samples (**Figures 3B** and **Supplementary Figure 4A**). Phylum level variation in different sampling sites of OSCC showed a higher abundance of *Bacteroidetes* (FDR-corrected *p* = 0.0002) and *Fusobacteria* (FDR-corrected *p* = 0.0007) in tumor site compared to contralateral healthy sites. *Actinobacteria* (FDR-corrected *p* = 0.0012) showed a reverse trend in aforementioned sample sites (**Figure 3C** and **Supplementary Figure 4A**). A similar pattern of variation of *Bacteroidetes* and *Fusobacteria* in healthy and OSCC samples reemphasize the possible deterministic shift of healthy oral microbiome to a distinct composition when subjected to an environmental stress which was smokeless tobacco consumption in this case.

### Higher Abundance of *Streptococcus* in Smokeless-Tobacco-Non-consuming Healthy Samples and Contralateral Healthy Site of OSCC Samples

A core microbiome analysis was performed considering genera with >1% abundance in all healthy and OSCC (100%) samples. Considering all 196 samples, *Streptococcus*, *Rothia*, *Granulicatella*, *Gamella*, and *Veilonella* constitute the core genera in the Indian cohort. Among these, *Granulicatella* and *Veilonella* were observed to be significantly lower in the healthy samples compared to the OSCC samples, whereas *Streptococcus* showed an opposite trend (**Figure 3D**; **Supplementary Table 4**). The core healthy oral microbiome in an Indian cohort (94 samples) comprised of *Streptococcus*, *Rothia*, *Gemella*, *Veillonella*, and *Granulicatella*. Boruta and LEfSe analysis revealed nine significantly discriminating genera based on smokeless tobacco consumption in healthy samples (**Supplementary Figures S5A, B**). *Streptococcus* was highly abundant in smokeless-tobacco non-consuming healthy samples compared to smokeless-tobacco-consuming healthy samples (Wilcoxon rank-sum test, *p* = 0.001). *Gemella* and *Haemophilus* were also having similar trends among healthy samples (Wilcoxon rank-sum test, *p* = 0.01 and 0.05, respectively). *Prevotella*, *Porphyromonas*, *Leptotrichia*, and *Fusobacterium* showed reverse trend among healthy samples (**Figure 3E**).

Out of 102 OSCC samples, *Porphyromonas, Actinomyces, Prevotella, Rothia, Capnocytophaga, Granulicatella, Gemella, Streptococcus, Veillonella, Selenomonas, Fusobacterium, Neisseria, Campylobacter *and* Haemophilus* were present among core OSCC oral microbiome in Indian cohort. Seven genera among them were significantly discriminating based on sampling site (tumor and contralateral healthy site). Interestingly, *Streptococcus* is highly abundant in the contralateral healthy site compared to tumor site (Wilcoxon rank-sum test, p = 0.001). On the other hand, *Staphylococcus, Capnocytophaga, Fusobacterium, *and* Campylobacter* were showing an opposite trend (**Figure 3F**). LEfSe analysis also revealed the differential abundance of *Capnocytophaga, Fusobacterium *and* Campylobacter* in tumor site using both Greengenes and SILVA databases (**Supplementary Figures S5C, D**; **S6**). Considering the higher abundance of *Streptococcus* in healthy subjects, smokeless-tobacco-non-consuming healthy samples and contralateral healthy site of OSCC subjects, *Streptococcus* appears as a marker in healthy oral site in the Indian cohort.

### Differentially Abundant Genera in Healthy and OSCC Oral Microbiome and Their Comparison With Taxonomic Composition of Tobacco Products

**Figure 4:**
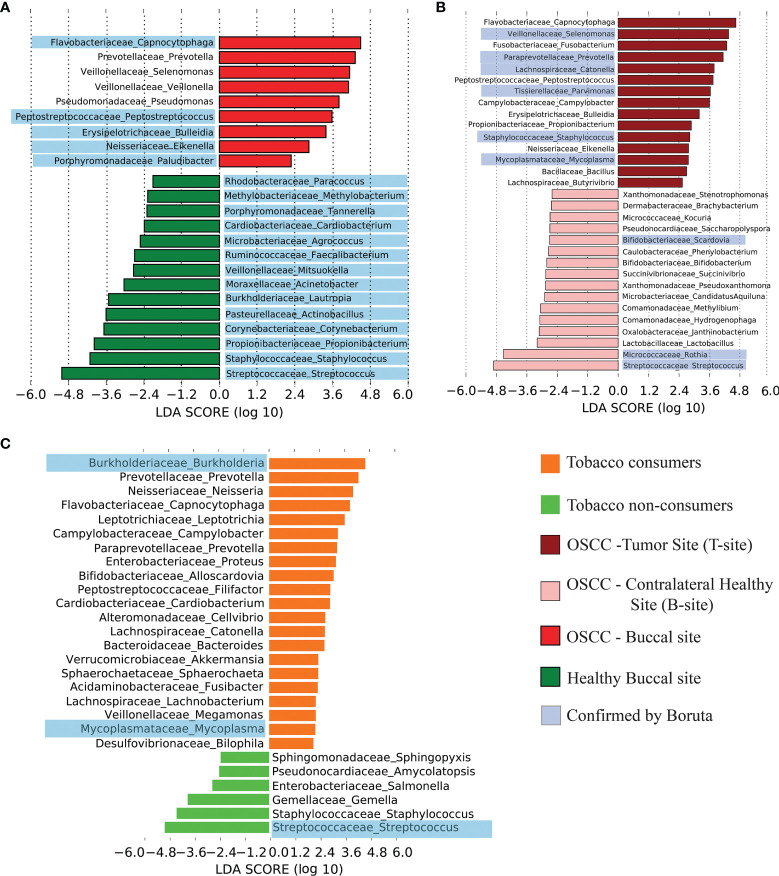
Differentially abundant genera in healthy and OSCC oral microbiome. **(A)** Differentially abundant genera in healthy and OSCC samples and corresponding linear discriminant analysis (LDA) score using LEfSe. The discriminating genera reported by the analysis using Boruta were highlighted in blue color. **(B)** Differentially abundant genera in the tumor site and contralateral healthy buccal site of OSCC samples and corresponding LDA score using LEfSe. The discriminating genera reported by the analysis using Boruta were highlighted in blue color. **(C)** Differentially abundant genera in smokeless-tobacco-consuming and non-consuming healthy samples and corresponding LDA score using LEfSe. The discriminating genera reported by the analysis using Boruta were highlighted in blue color.

As per the observations from beta diversity analysis, four major groups of samples (healthy smokeless-tobacco-non-consuming, healthy smokeless-tobacco-consuming, OSCC tumor site and OSCC contralateral buccal site) were considered for further analysis. LEfSe and Boruta were used to identify the differentially enriched genera in all the groups. Analysis of differentially abundant genera in healthy and OSCC buccal sites showed significantly higher abundance of *Capnocytophaga*, *Prevotella*, *Selenomonas*, *Pseudomonas*, *Veillonella Peptostreptococcus*, *Bulleidia*, *Eikenella* and *Paludibacter* in the OSCC patients using LEfSe. Among these, *Capnocytophaga*, *Peptostreptococcus*, *Bulleidia*, *Eikenella*, and *Paludibacter* were also confirmed to be the most discriminatory by Boruta (**Figure 4A**). Several genera including *Streptococcus*, *Staphylococcus*, *Propionibacterium*, *Corynebacterium*, *Actinobacillus*, *Lautropia*, *Acinetobacter*, *Mitsuokella*, *Faecalibacterium*, *Agrococcus*, *Cardiobacterium*, *Tannerella*, *Methylobacterium*, and *Paracoccus* showed enrichment in the healthy individuals in both LEfSe and Boruta analysis (**Supplementary Figure S7**).

Differentially abundant genera in tumor site compared to contralateral healthy buccal sites were also explored to analyze the microbial composition at tumor site. *Capnocytophaga*, *Selenomonas*, *Fusobacterium*, *Prevotella*, *Catonella*, *Peptostreptococcus*, *Parvimonas*, *Campylobacter*, *Bulleidia*, *Propionobacterium*, *Eikenella*, etc. were differentially abundant in tumor site compared to contralateral healthy site (**Figure 4B**). Most of these genera are already shown to be abundant in OSCC compared to healthy buccal site (**Supplementary Figures S7**, **S8**).

The effect of smokeless tobacco consumption in oral microbiome composition was also investigated by identifying differentially abundant genera in oral buccal site of smokeless-tobacco-consuming and non-consuming healthy individuals. As mentioned earlier, *Streptococcus* is one of the genera that showed significantly higher abundance in smokeless tobacco non-consumers that was identified by both LEfSe and Boruta analysis. In addition, *Staphylococcus*, *Gemella*, *Salmonella*, *Amycolaptosis*, and *Shingopyxis* were also differentially abundant in smokeless tobacco non-consumers (**Figure 4C**). *Burkholderia*, *Prevotella*, *Neisseria*, *Capnocytophaga*, *Leptotrichia*, *Campylobacter*, *Proteus*, *Alloscardovia*, *Filifacter*, *Cardiobacterium*, *Cellvibrio*, *Catonella*, *Bacteroides*, *Akkermansia*, *Sphaerochaeta*, *Fusibacter*, *Lachnobacterium*, *Megamonas*, *Mycoplasma*, and *Bilophila* were differentially abundant in smokeless-tobacco-consuming healthy individuals (**Supplementary Figure S9**). Differentially abundant genera in dental samples were also analysed separately and showed similar microbiome composition as found in buccal site of healthy and OSCC samples (**Supplementary Figures S10**, **S11**).

We also examined the microbiome associated with tobacco products and the microbiome in different groups of oral sample sites (healthy buccal site, OSCC buccal site, TC-H, NTC-H, OSCC-T-site, OSCC-B-site). Interestingly, most of the genera present in tobacco products were found to overlap (in terms of presence and absence) with differentially abundant genera of OSCC buccal site (compared to healthy buccal controls), smokeless-tobacco-consuming healthy buccal site (compared to tobacco non-consumers), and OSCC tumor site (compared to contralateral healthy buccal site of OSCC samples) (**Supplementary Figure S12A**). Twenty out of 22 (90.9%) differentially abundant genera in OSCC buccal site were found to be present among the top 50 genera in tobacco products, whereas only 12 out of 38 (31.57%) differentially abundant genera in the healthy buccal site were present among the top 50 genera in tobacco products. Furthermore, we examined the relative abundance of the above genera in tobacco products. *Capnocytophaga*, *Prevotella*, *Selenomonas*, *Actinomyces*, *Veillonella*, *Peptostreptococcus*, *Granulicatella*, *Campylobacter*, *Pseudomonas*, and *Catonella* were among the top 10 genera in tobacco products that were also among the differentially abundant genera in OSCC buccal site (**Supplementary Figure S12C**). *Streptococcus* was highly abundant (between 0.25 to 0.32) in tobacco products. Comparatively higher abundance of *Corynebacterium* in “T-GU” was also observed. The remaining six genera were below the relative abundance criteria of 0.05 (**Supplementary Figure S12B**).

### Differentially Abundant Species

**Figure 5:**
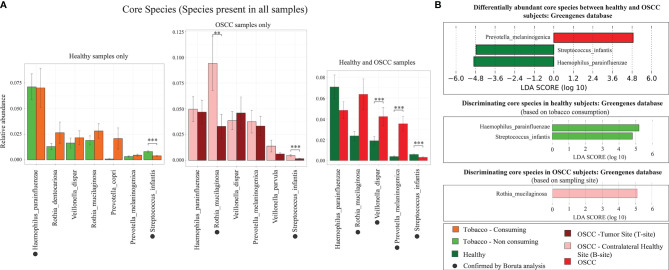
Differentially abundant species. **(A)** Relative abundance of core microbial species in smokeless-tobacco-consuming and non-consuming healthy samples, and tumor site and contralateral healthy buccal site of OSCC samples. The significance levels were indicated based on Wilcoxon rank-sum test. Species indicated by black filled dots are discriminating core microbial species identified by Boruta. **(B)** Differentially abundant core microbial species in smokeless-tobacco-consuming and non-consuming healthy samples, and tumor site and contralateral healthy buccal site of OSCC samples. LDA score using LEfSe was also indicated in this figure. **p < 0.01; ***p < 0.001.

The hypervariable regions in 16S rRNA gene such as the V3 region is capable of identifying the genera, although it has limitations to adequately discriminate between species (Ranjan et al., 2016). However, we attempted the species identification using both Greengenes and SILVA databases. Analysis based on Greengenes database resulted in assignment of ~30% OTUs to species level, and analysis using SILVA database reported most of the OTUs as unclassified species (assignment till genus level). Species level analysis using LEfSe and Boruta indicated that *Rothia musilaginosa*, *Veillonella dispar*, *Prevotella melaninogenica*, and *Streptococcus infantis* are differentially abundant core oral species (present in all oral samples) (**Figures 5A, B**).

The species level analysis indicated that *V. dispar* is significantly enriched in OSCC patients (**Figure 5A**). It is also differentially abundant in healthy smokeless tobacco consumers and tumor site of OSCC patients compared to their respective controls. Evidence from previous studies showed that *V. dispar* was able to distinguish current smokers and never smokers with the highest performance using the random forest classifier (Sato et al., 2020), which indicates the possibility of considering it as a marker species for smokeless tobacco consumption/OSCC. Our analysis in oral microbiome found significant difference of *Rothia mucilaginosa* between healthy and OSCC buccal samples in terms of relative abundance (higher abundance in OSCC) by Boruta (**Figure 5A**). In addition, it was found to be highly abundant in smokeless-tobacco-consuming healthy samples compared to non-consumers and highly abundant in contralateral healthy buccal site of OSCC samples compared to tumor site. Thus, these two marker species can help in early diagnosis of oral microbiome dysbiosis in healthy and OSCC samples.

### Are These Marker Genera Universal?

**Figure 6:**
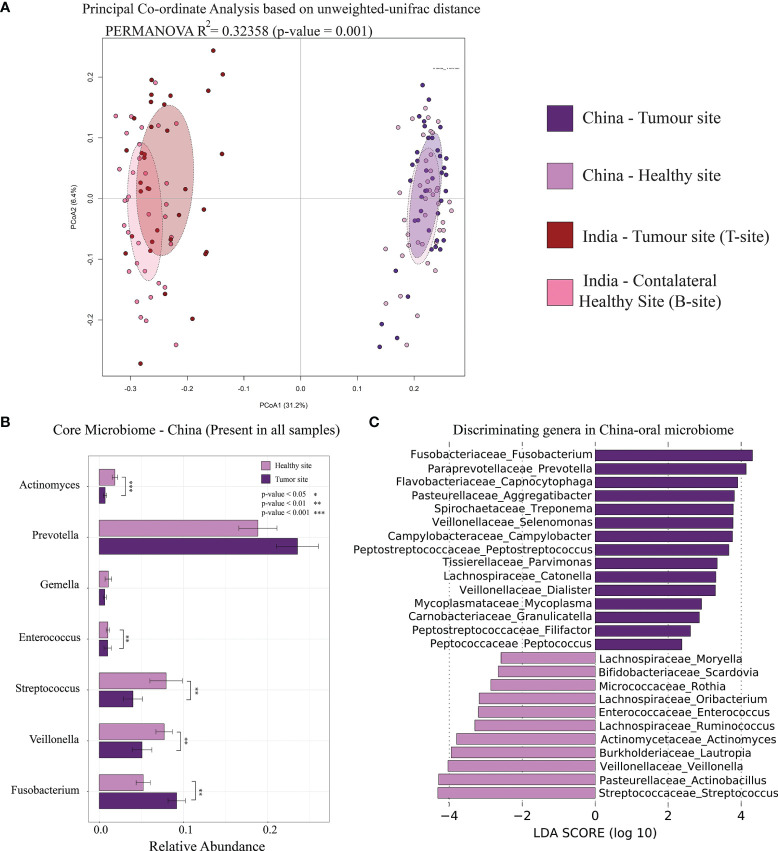
Comparative analysis of Indian and Chinese oral microbiome of OSCC samples. **(A)** Principal coordinate analysis of Indian and Chinese OSCC samples based on intersample unweighted-UniFrac distance. **(B)** Relative abundance of core-oral microbiome (genus level) in Chinese samples. The significance levels were indicated based on Wilcoxon rank-sum test. **(C)** Differentially abundant microbial genera in the tumor site and healthy site of OSCC samples. LDA score using LEfSe were also indicated in this figure.

We have carefully chosen a publicly available oral microbiome dataset of OSCC patients similar to the sampling site, sample collection procedure, and sequencing platform used in our study (Zhao et al., 2017). Out of the 80 oral microbiome samples from OSCC patients (V4–V5 region of 16S rRNA gene) from this study, 40 were collected from OSCC tumor site and 40 from anatomically opposite healthy buccal site. Our objective to carry out this analysis was to check if the marker genera found in our study are universally the same. Initial PCoA analysis based on unweighted-UniFrac distance revealed a clear separation of samples between Indian and Chinese datasets (**Figure 6A**). These distinct clustering may be attributed to different environment, dietary pattern, and also possibly due to the different regions of 16S rRNA used for the analysis. Core microbiome analysis of this dataset indicated *Prevotella* as the most abundant oral genus and was also differentially abundant in the tumor site compared to that in the healthy buccal site. *Streptococcus* was differentially abundant in the healthy site, and *Fusobacterium* was dominant at the tumor site (**Figure 6B**). *Fusobacterium*, *Capnocytophaga*, *Selenomonas*, *Prevotella*, *Peptostreptococcus*, *Parvimonas*, *Campylobacter*, and *Mycoplasma* were dominant in the tumor site of both Indian and Chinese cohort (**Figure 6C**).

### Correlation Between Microbial Genera and Metabolic Pathways in Oral Microbiome

Co-occurrence of genera in healthy (both smokeless-tobacco-consuming and non-consuming) and OSCC samples (both T-site and B-site) were examined separately. All significant positive correlations >0.5 were used for this analysis. In smokeless-tobacco-consuming healthy samples, there were strong intercorrelations between *Prevotella*, *Selenomonas*, *Leptotrichia*, *Capnocytophaga*, *Catonella*, *Neisseria*, *Campylobacter*, and *Fusobacterium* (**Supplementary Tables 5**, **6**). Interestingly, they were differentially abundant in OSCC and in smokeless-tobacco-consuming healthy samples. Intercorrelation between aforementioned genera was lesser in smokeless-tobacco-consuming healthy samples. The correlation landscape changed drastically in OSCC samples with lesser number of genera and connections. Metabolic pathways identified based on PICRUSt analysis that were differentially abundant in healthy and OSCC samples were also examined. Lipopolysaccharide (LPS) biosynthesis pathway was positively correlated with the differentially abundant genera in OSCC samples. Carbohydrate metabolism and xylene degradation were positively correlated with *Streptococcus*, *Staphylococcus*, and *Agrococcus* that were differentially abundant in healthy oral microbiome, whereas strong negative correlation was observed with *Prevotella*, *Capnocytophaga*, and *Eikenella* that were differentially abundant in OSCC and smokeless-tobacco-consuming healthy samples. Pores and ion channels, D-glutamine and D-glutamate metabolism, and amino-acid-related enzymes showed positive correlation with *Capnocytophaga* and *Eikenella* (**Supplementary Figure S13A**). A similar pattern was found in correlation of metabolic pathways with TC-H and NTC-H samples (**Supplementary Figure S13B**, **Supplementary Table S7**).

### Metabolomic Analysis

UPLC-MS-based analysis of oral metabolome of healthy and OSCC samples exhibit clear separation in principle coordinate analysis (**Supplementary Figure S14A**). Sphinganine, Nedocromil sodium, Estrane, Procainamide, 1-Nitrosonaphthalene, Tolmetin, glutamine, histidine, Azelaic acid, etc. were positively associated with OSCC samples (**Supplementary Figures S14B, C**; **Supplementary Text**). Dihydrosphingosine (sphinganine) was found to be associated with OSCC samples and smokeless-tobacco-consuming healthy samples. We have also calculated the Spearman correlation between the abundance of microbial genera and the metabolome of each group of samples and did not observe any significant correlation between them. The probable reason behind this observation can be the small sample size used for metabolome analysis.

## Discussion

Oral cancer is highly prevalent in South Asian countries including India, Bangladesh, Sri Lanka, and Pakistan and is the third most common and fifth leading cause of cancer-associated deaths in this region (Ranganathan et al., 2004; Ferlay et al., 2015; Amarasinghe et al., 2018). The use of tobacco, areca nut (Sinor et al., 1990; Murti et al., 1995), and betel quid (Hernandez et al., 2017), which are the major ingredients of *gutkha* and *pan masala*, have been known for their strong inflammatory and carcinogenic effects on humans. At the clinical level, the association between oral inflammation and induction of oral cancer has been well established (Feller et al., 2013; Niklander, 2021). However, an alteration in microflora or selective growth of certain species or strains also plays a crucial role in carcinogenicity (Irfan et al., 2020). The role of microbiome in the development of oral cancer can be explained by bacterial simulation, pathogenesis, and production of potential carcinogens. (Zhang et al., 2018; Karpiński, 2019).

Therefore, the key focus of this study was to decipher the dysbiosis in the oral microflora of healthy and OSCC patients (94 healthy and 102 OSCC samples) due to the consumption of smokeless-tobacco-based products. This study revealed the smokeless-tobacco-associated metagenomic community using an unbiased approach to capture site-specific differences in the oral microbiome composition. Inclusion of both smokeless-tobacco-consuming and non-consuming individuals in comparable proportions and independent analysis of the microbiome of tobacco products contributed to elucidate the role of tobacco consumption in the oral microbiome composition.

Higher richness and diversity were observed in the oral microbiome of healthy samples compared to OSCC samples. Interestingly, healthy smokeless tobacco consumers showed higher species richness and evenness compared to non-consumers. A similar observation was reported in a study conducted in a Middle Eastern population consuming Middle Eastern tobacco products like dokha and shisha (Vallès et al., 2018). Another study also reported that microbiome in smokers exhibit a significantly higher Shannon diversity index than that in non-smokers (Mason et al., 2015). Health status, smokeless tobacco consumption, and sampling site were significantly associated with intersample variation, and in particular, the health status was found to be the major driving force of oral microbiome variation compared to the other covariates. Intersample variation in healthy oral microbiome was significantly associated with smokeless tobacco consumption status, whereas no significant association of smokeless tobacco consumption was observed in oral cancer samples. Intersample variation of OSCC oral microbiome was significantly associated with site of sampling, i.e., tumor site or buccal site far distant from the tumor site.

**Figure 7:**
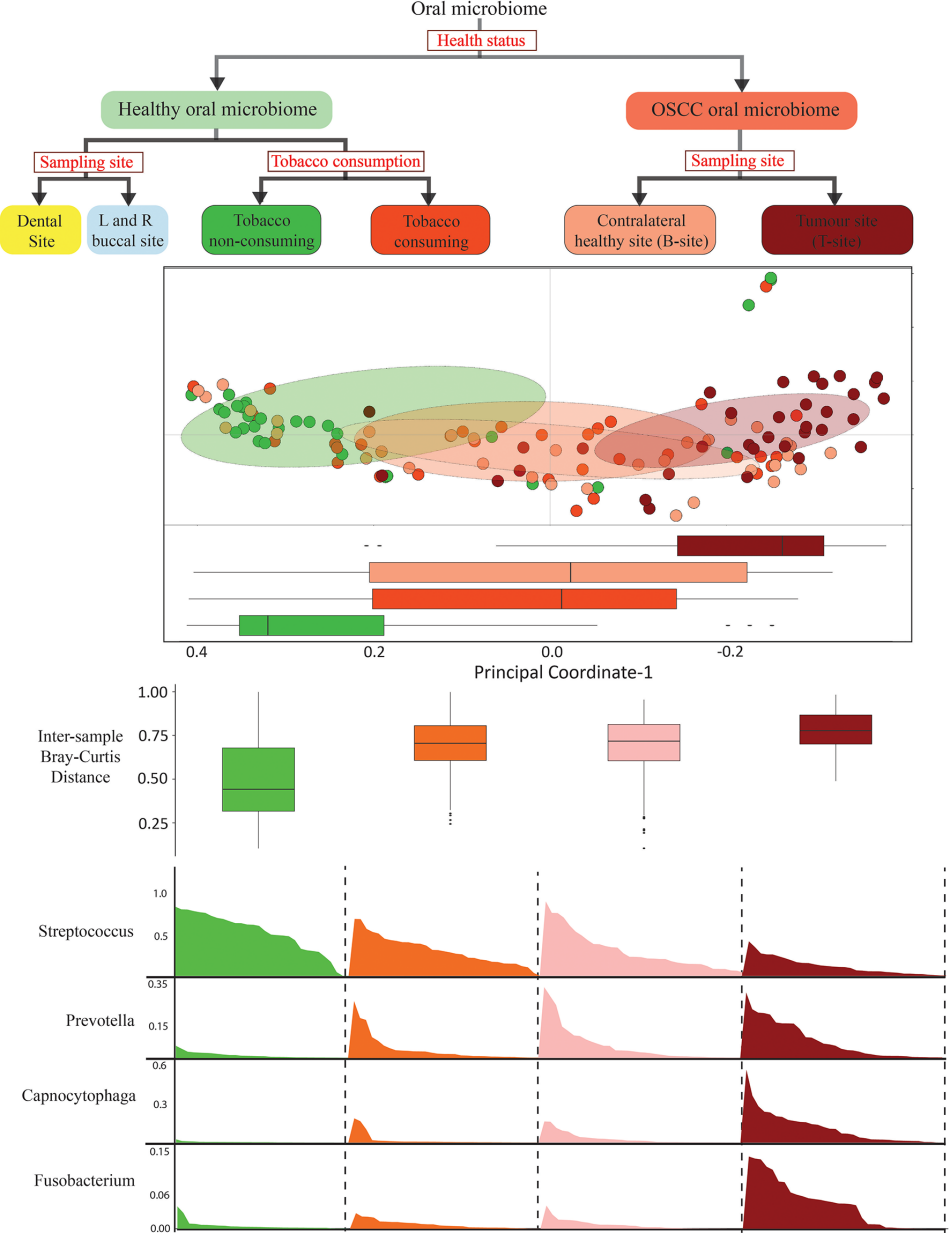
Schematic representation of the design and outcome of this study.

The beta-diversity was observed to be the lowest in healthy smokeless tobacco non-consumers and the highest in oral tumor site of OSCC samples, whereas the healthy smokeless tobacco consumers and the contralateral buccal site of OSCC samples showed intermediate values. In addition, a remarkable similarity between healthy smokeless tobacco consumers’ samples and the contralateral buccal site of OSCC samples was observed based on intersample distances in both buccal and dental microbiome. Furthermore, principal coordinate analysis suggest a clear transition of oral microbiome from healthy smokeless tobacco non-consumers to OSCC oral tumor site samples with healthy smokeless tobacco consumers samples, and the contralateral buccal site of OSCC samples showed intermediate values with a significant overlap. These observations align well with the concept of “Anna Karenina principle” for animal microbiomes (Zaneveld et al., 2017), which refer to a more variable microbial community composition in dysbiotic individuals compared to healthy individuals. These observations indicate a deterministic shift of healthy oral microbiome during environmental stress conditions, like tobacco consumption, to a more dispersed microbial community composition in tobacco consuming individuals that is similar to the microbiome associated with OSCC buccal site (**Figure 7**).

Analysis of differentially abundant microbial phyla in these four groups of samples revealed a significantly higher abundance of *Bacteroidetes* and *Fusobacteria* in the tumor site of OSCC samples compared to the contralateral healthy site, which can be interpreted as an inflammation-associated change in microbiome composition (Li and Ma, 2020). Significantly higher abundance of *Bacteroidetes* in OSCC samples compared to healthy samples supported this explanation. Interestingly, significantly higher abundance of *Bacteroidetes* and *Fusobacteria* in healthy smokeless tobacco consumers compared to non-consumers confirmed that the change in healthy oral microbiome due to smokeless tobacco consumption is comparable to inflammation-associated microbiome. In addition, depletion of *Firmicutes* in OSCC samples compared to healthy samples and smokeless-tobacco-consuming healthy samples compared to non-consuming healthy samples reaffirmed the resemblance of oral microbiome of healthy smokeless tobacco consumers with inflammation-associated oral microbiome. A significantly higher abundance of *Prevotella*, *Capnocytophaga*, *Leptotrichia*, *Fusobacterium*, etc. was revealed in smokeless-tobacco-consuming healthy samples and tumor site of OSCC samples. Similar observations in comparative analysis using OSCC oral microbiome samples from the Chinese dataset indicated that significantly higher abundance of the above-mentioned genera can be considered as potential microbiome markers for inflammation-associated microbiome. Earlier studies identified *Fusobacteria* to be metabolically hyperactive in the oral community of OSCC patients (Yost et al., 2018), echoing the previous findings in colorectal cancer (Castellarin et al., 2012; Kostic et al., 2012). Association of several *Prevotella* species such as *Prevotella intermedia* and *Prevotella nigrescens* with oral inflammations like periodontitis has been reported (Mättö et al., 1997; Deng et al., 2017). Apparently, smokeless tobacco consumption creates a microenvironment that selects for a large and specific group of microorganisms. Positive association of LPS biosynthesis pathways-related proteins with differentially abundant genera in OSCC and smokeless-tobacco-consuming healthy samples further confirms the differential abundance of Gram-negative marker genera in OSCC microbiomes. Previous reports established that smokers showed higher abundances of anaerobes and lower levels of aerobes when compared with non-smokers, and a similar trend was observed in OSCC samples compared to that in healthy samples (Mason et al., 2015). This change in microbiome composition could be one of the mechanisms by which smokeless tobacco consumption increases the risk for oral cancer. In summary, the similarity in oral microbiome composition of healthy smokeless tobacco consumers and OSCC tumor site indicates a possible role of tobacco consumption in transition of healthy oral microbiome to an inflammation-associated microbiome.

Arecoline, a component of areca nut, has been shown to induce several pro-carcinogenic changes, including the production of nitrosamines and reactive oxygen species (Moutasim et al., 2011), and increased expression of inflammatory cytokines, including tumor necrosis factor-α, interleukin-6, interleukin-8, and interleukin-1-β (Chang et al., 2009). Additionally, nitrosamines derived from betel quid and tobacco mediating oxidation of thiol group of antioxidants such as glutathione-S-transferase, superoxide dismutase (SOD), glutathione reductase, glutathione peroxidase (GPx), and catalase (CAT) were reported (Shiu and Chen, 2004) to induce changes in oral microbiome communities contributing to oral carcinogenesis (Lee et al., 2017; Yang et al., 2018). Although the carcinogenic properties of betel nut and other tobacco product components has been known, the microbiome composition of tobacco products was required to be analyzed along with a comparison of oral microbiome of healthy and OSCC samples, which was performed in this study. The analysis revealed that *Capnocytophaga*, *Prevotella*, *Selenomonas*, *Actinomyces*, *Veillonella*, *Peptostreptococcus*, *Granulicatella*, *Campylobacter*, *Pseudomonas*, and *Catonella* were among the top 10 genera in tobacco products, and these were differentially abundant in OSCC samples. These results provide leads for further studies to understand the role of microbiome composition of tobacco products in altering the oral microbiome.

Another interesting observation from this study is the role of *Streptococcus* genus as a marker of healthy oral microbiome. Significantly higher abundance of *Streptococcus* was observed in healthy individuals compared to OSCC samples, non-consumers of smokeless tobacco compared to smokeless tobacco consumers among healthy samples, and contralateral healthy site compared to tumor site in OSCC samples. *Streptococci* are among the early colonizers of oral microbiome with diverse acidogenic and aciduric properties (Zhu et al., 2018). Previous reports of significant elevation or reduction in the relative abundance of common oral bacteria including *Streptococcus* in betel nut chewers are consistent with our study (Aas et al., 2005; Bik et al., 2010). Lower abundance of *Streptococcus* genera in smokeless tobacco consumers observed in this study can be explained by the antibacterial properties of betel nut/tobacco components. Analysis of differentially abundant species indicated significantly higher abundance of *S. infantis* in smokeless tobacco non-consumers compared to that in smokeless tobacco consumers and contralateral buccal site of OSCC samples compared to that in the OSCC tumor site. The growth of common *Streptococcus* species, in particular *Streptococcus intermedius*, *Streptococcus anguinis*, and *Streptococcus mutans* from saliva are shown to be suppressed by prolonged exposure to the aqueous extracts of betel nut such as tannic acid (De Miranda et al., 1996). In murine models, an anaerobic streptococcal species, *Streptococcus anginosus*, is shown to induce the synthesis of inflammatory cytokines and NO (Sasaki et al., 2001) signifying potential mechanisms of carcinogenesis. Since previous studies showed association of different *Streptococcus* species with healthy and inflammation conditions, more detailed species/strain level analysis is required to capture species level differences.

Using the V3 hypervariable region of 16S rRNA genes, only ~30% species-level annotation could be achieved that showed *R. mucilaginosa*, *P. melaninogenica*, and *V. dispar* to be differentially abundant in OSCC samples compared to that in healthy samples. Previous studies have reported the association of *P. melaninogenica* with oral cancer (Mager et al., 2005). UPLC-MS-based analysis of oral metabolome of healthy and OSCC samples showed several metabolites that were positively associated with OSCC samples. In particular, dihydrosphingosine (sphinganine) was found to be associated with OSCC samples and smokeless-tobacco-consuming healthy samples. The observed lack of correlation between the abundance of microbial genera and the metabolome of each group of samples was perhaps due to the small sample size used for the metabolome analysis, and more insights may emerge from the analysis of larger cohorts.

In summary, this study provides the initial insights on the smokeless-tobacco-associated oral microbiome and oral cancer. This study emphasizes that the oral microbiome of healthy individuals is significantly affected by smokeless tobacco consumption. A possible role of smokeless tobacco consumption in transition of healthy oral microbiome to inflammation-associated oral microbiome was apparent with a deterministic shift of oral microbiome composition in healthy to OSCC samples with intermediate overlap between tobacco consuming healthy and contralateral buccal site of OSCC samples. This aligns well with the concept of “Anna Karenina principle” for animal microbiomes (Zaneveld et al., 2017), which refers to a more variable microbial community composition in dysbiotic individuals compared to healthy individuals—paralleling Leo Tolstoy’s dictum that “all happy families look alike; each unhappy family is unhappy in its own way” (Zaneveld et al., 2017). Significantly higher abundance of *Streptococcus* emerged as a marker for the healthy oral microbiome, which was also supported by comparative analysis of other OSCC microbiome cohorts. Taken together, the abundance of marker genera in healthy and inflammation-associated oral microbiomes reaffirms the potential impact of smokeless tobacco consumption in the dysbiosis of oral microbiome (**Figure 7**).

However, the present amplicon-based study was performed on a limited number of samples from an important geographical region. Therefore, large-scale and longitudinal metagenomic studies on cohorts from different geographical regions are much needed to understand the underlying mechanisms of microbiome-associated dysbiosis and carcinogenicity. To harness the potential of oral microbiome in developing novel diagnostic and therapeutic methods for oral cancer caused by tobacco consumption, in-depth studies combining metagenomic, transcriptomic, and metabolomic approaches will be highly relevant for countries including India and other South Asian countries that show a large prevalence of oral cancer cases plausibly due to the excessive consumption of smokeless tobacco products.

## Data Availability Statement

The datasets presented in this study can be found in online repositories. The names of the repository/repositories and accession number(s) can be found below: https://www.ncbi.nlm.nih.gov/, accession ID: PRJNA789915.

## Ethics Statement

The studies involving human participants were reviewed and approved by Institute Ethics Committee (IEC), Indian Institute of Science Education and Research (IISER), Bhopal, India. The patients/participants provided their written informed consent to participate in this study.

## Author Contributions

VKS conceived the work. VPPK and RS have contributed equally to this work and share first authorship. VKS, RS, SG, AS and VPPK participated in the design of the study. RS, SG, PW, SVG performed the sample collection in collaboration with AS. RS designed the experimental protocols and performed sample processing, DNA extraction, library preparation and sequencing work with SVG, PW and AS. VPPK carried out 16S rRNA, comparative metagenomic data analysis, statistical analysis, data visualization, and prepared all the main figures. ASK and VPPK carried out the metabolomic data analysis. VPPK, RS, AKS and VKS carried out the interpretation of results. VPPK, RS, AS, AKS and VKS drafted the final manuscript. All authors contributed to the article and approved the submitted version.

## Funding

This work was supported by the intramural funding received from IISER Bhopal, Madhya Pradesh, India.

## Conflict of Interest

The authors declare that the research was conducted in the absence of any commercial or financial relationships that could be construed as a potential conflict of interest.

## Publisher’s Note

All claims expressed in this article are solely those of the authors and do not necessarily represent those of their affiliated organizations, or those of the publisher, the editors and the reviewers. Any product that may be evaluated in this article, or claim that may be made by its manufacturer, is not guaranteed or endorsed by the publisher.

## Supplementary Material

Supplementary Figure 1 Alpha-diversity analysis of healthy and OSCC oral microbiome considering the smokeless tobacco consumption status and sampling sites. **(A)** Box plots representing alpha-diversity of healthy and OSCC samples (n=196) based on Shannon, Simpson and Chao indexes. **(B)** Box plots representing alpha-diversity of smokeless tobacco consuming and non-consuming healthy samples (n=94) based on Shannon, Simpson and Chao indexes. **(C)** Box plots representing alpha-diversity of smokeless tobacco consuming and non-consuming OSCC samples (n=102) based on Shannon, Simpson and Chao indexes. **(D)** Box plots representing alpha-diversity of left-, right- buccal samples and dental samples in healthy (n=94) individuals based on Shannon, Simpson and Chao indexes. **(E)** Box plots representing alpha-diversity of samples from tumour site, contralateral healthy site and dental sites in OSCC (n=102) patients based on Shannon, Simpson and Chao indexes.

Supplementary Figure 2 Inter-sample diversity (beta-diversity) analysis of healthy and OSCC oral microbiome considering the smokeless tobacco consumption status and sampling sites. **(A)** Principal Coordinate Analysis of healthy and OSCC oral microbiome (n=196) based on inter-sample unweighted-unifrac distance. **(B)** Principal Coordinate Analysis of smokeless tobacco consuming and non-consuming healthy oral microbiome (n=94) based on inter-sample unweighted-unifrac distance. **(C)** Principal Coordinate Analysis of healthy oral microbiome (n=94) of left-, right- buccal sites and dental sites based on inter-sample unweighted-unifrac distance. **(D)** Principal Coordinate Analysis of smokeless tobacco consuming and non-consuming OSCC oral microbiome (n=102) based on inter-sample unweighted-unifrac distance. **(E)** Principal Coordinate Analysis of smokeless tobacco consuming and non-consuming OSCC oral microbiome (n=102) based on inter-sample Bray-Curtis distance. **(F)** Principal Coordinate Analysis of oral microbiome of tumour site, contralateral healthy site and dental site of OSCC (n=102) samples based on inter-sample unweighted-unifrac distance.

Supplementary Figure 3 Relative abundance of bacterial phyla and genera in healthy and OSCC oral microbiome. **(A)** Relative abundance of bacterial phyla in healthy and OSCC oral microbiome (n=196). **(B)** Relative abundance of bacterial genera in healthy oral microbiome (n=94). **(C)** Relative abundance of bacterial genera in OSCC oral microbiome (n=102).

Supplementary Figure 4 Comparison of relative abundance of bacterial phyla and genera in smokeless tobacco consuming and non-consuming healthy samples. **(A)** Relative abundance of bacterial phyla in smokeless tobacco consuming and non-consuming healthy oral microbiome (n=94). **(B)** Relative abundance of bacterial genera in smokeless tobacco consuming and non-consuming healthy oral microbiome (n=94).

Supplementary Figure 5 Differentially abundant genera in healthy and OSCC samples considering smokeless tobacco consumption status and sampling sites into account. **(A)** Core genera that are differentially abundant in smokeless tobacco-consuming and non-consuming healthy oral microbiome. Corresponding LDA score for each genus reported by LEfSe were indicated in the figures. Taxonomic annotation of OTUs were carried out using Greengenes database. **(B)** Core genera that are differentially abundant in smokeless tobacco-consuming and non-consuming healthy oral microbiome. Corresponding LDA score for each genus reported by LEfSe were indicated in the figures. Taxonomic annotation of OTUs were carried out using SILVA database. **(C)** Core genera that are differentially abundant in tumour site and contralateral healthy buccal site of OSCC microbiome. Corresponding LDA score for each genus reported by LEfSe were indicated in the figures. Taxonomic annotation of OTUs were carried out using Greengenes database. **(D)** Core genera that are differentially abundant in tumour site and contralateral healthy buccal site of OSCC microbiome. Corresponding LDA score for each genus reported by LEfSe were indicated in the figures. Taxonomic annotation of OTUs were carried out using SILVA database. **(E)** Relative abundance of core-genera (annotated using SILVA) in smokeless tobacco consuming and non-consuming healthy samples. **(F)** Relative abundance of core-genera (annotated using SILVA) in tumour site and contralateral healthy site of OSCC samples.

Supplementary Figure 6 Relative abundance of bacterial phyla and genera in OSCC microbiome. **(A)** Relative abundance of bacterial phyla in dental site, tumour site and contralateral healthy site. **(B)** Relative abundance of bacterial genera in dental site, tumour site and contralateral healthy site.

Supplementary Figure 7 Differentially abundant bacterial genera in healthy and OSCC buccal site. **(A, B)** Relative abundance of differentially abundant bacterial genera in OSCC and healthy buccal site. These genera were identified using LEfSe and Boruta.

Supplementary Figure 8 Differentially abundant bacterial genera in tumour site and contralateral healthy buccal site of OSCC samples. **(A, B)** Relative abundance of differentially abundant bacterial genera in tumour site and contralateral healthy site of OSCC samples. These genera were identified using LEfSe and Boruta.

Supplementary Figure 9 Differentially abundant bacterial genera in smokeless tobacco-consuming and non-consuming healthy samples. **(A, B)** Relative abundance of differentially abundant bacterial genera in healthy oral microbiome of smokeless tobacco consumers and non-consumers. These genera were identified using LEfSe and Boruta.

Supplementary Figure 10 Differentially abundant bacterial genera in dental sites of healthy and OSCC samples. **(A)** Genera that are differentially abundant in smokeless tobacco-consuming and non-consuming healthy dental microbiome. Corresponding LDA score for each genus reported by LEfSe were indicated in the figures. **(B)** Genera that are differentially abundant in smokeless tobacco non-consuming healthy dental microbiome and OSCC dental microbiome. Corresponding LDA score for each genus reported by LEfSe were indicated in the figures.

Supplementary Figure 11 Relative abundance of differentially abundant bacterial genera in healthy dental samples considering their smokeless tobacco consumption status. **(A, B)** Relative abundance of differentially abundant bacterial genera in dental microbiome of healthy smokeless tobacco-consuming and non-consuming samples. These genera were identified using LEfSe and Boruta.

Supplementary Figure 12 Analysis of microbiome composition in tobacco-product samples. **(A)** Examining the presence/absence of differentially abundant genera in smokeless tobacco consumers and non-consumers of healthy samples, tumour site and contralateral healthy buccal site of OSCC samples and top 50 core genera in tobacco products. **(B)** Relative abundance of genera differentially abundant in healthy buccal sites in tobacco products. **(C)** Relative abundance of genera differentially abundant in OSCC buccal sites in tobacco products.

Supplementary Figure 13 Correlation analysis of differentially abundant genera and pathways in healthy and OSCC samples. **(A)** Heatmap indicating the significant correlation (Spearman) between differentially abundant genera and pathways in healthy and OSCC samples. **(B)** Network plot indicating the significant correlation (Spearman) between differentially abundant genera and pathways in smokeless tobacco-consuming and non-consuming healthy samples.

Supplementary Figure 14 Metabolome Analysis. Metabolomic analysis of Healthy and OSCC samples. Due to limited number (four) of samples from the healthy smokeless tobacco-consuming group, the metabolomic differences due to smokeless tobacco consumption were difficult to conclude and hence, are not shown here. **(A)** Principal Coordinate Analysis based on the Bray-Curtis distance between healthy and OSCC samples **(B)** Heatmap representing the association (Spearman) between metabolites and oral metabolome samples. **(C)** Mean decrease in accuracy of each metabolites calculated based on RandomForest analysis.

Supplementary Table 1 Metadata of all healthy and OSCC samples considered for this analysis.

Supplementary Table 2 Number of sequenced reads per sample from each healthy and OSCC sample considered for analysis after quality filtration.

Supplementary Table 3 Evaluation of the effect of covariates in the microbiome composition. The table indicates results of PERMANOVA analysis performed using Bray-Curtis distances between samples and different covariates.

Supplementary Table 4 Core microbial genera in oral microbiome based on their presence in all samples (100%), 95% samples, 90% samples samples.

Supplementary Table 5 Correlation (Spearman) between genera in healthy (both TC-H and NTC-H) samples.

Supplementary Table 6 Correlation (Spearman) between genera in OSCC samples (both T-site and B-site).

Supplementary Table 7 Correlation (Spearman) between differentially abundant genera and pathways in healthy and OSCC samples.
