# Supplementary figures and images for: Assessing the Effect of Smokeless Tobacco Consumption on Oral Microbiome in Healthy and Oral Cancer Patients
Source: Front Cell Infect Microbiol. 2022 Mar 31;12:841465. doi: 10.3389/fcimb.2022.841465 (PMC9009303; doi:10.3389/fcimb.2022.841465)

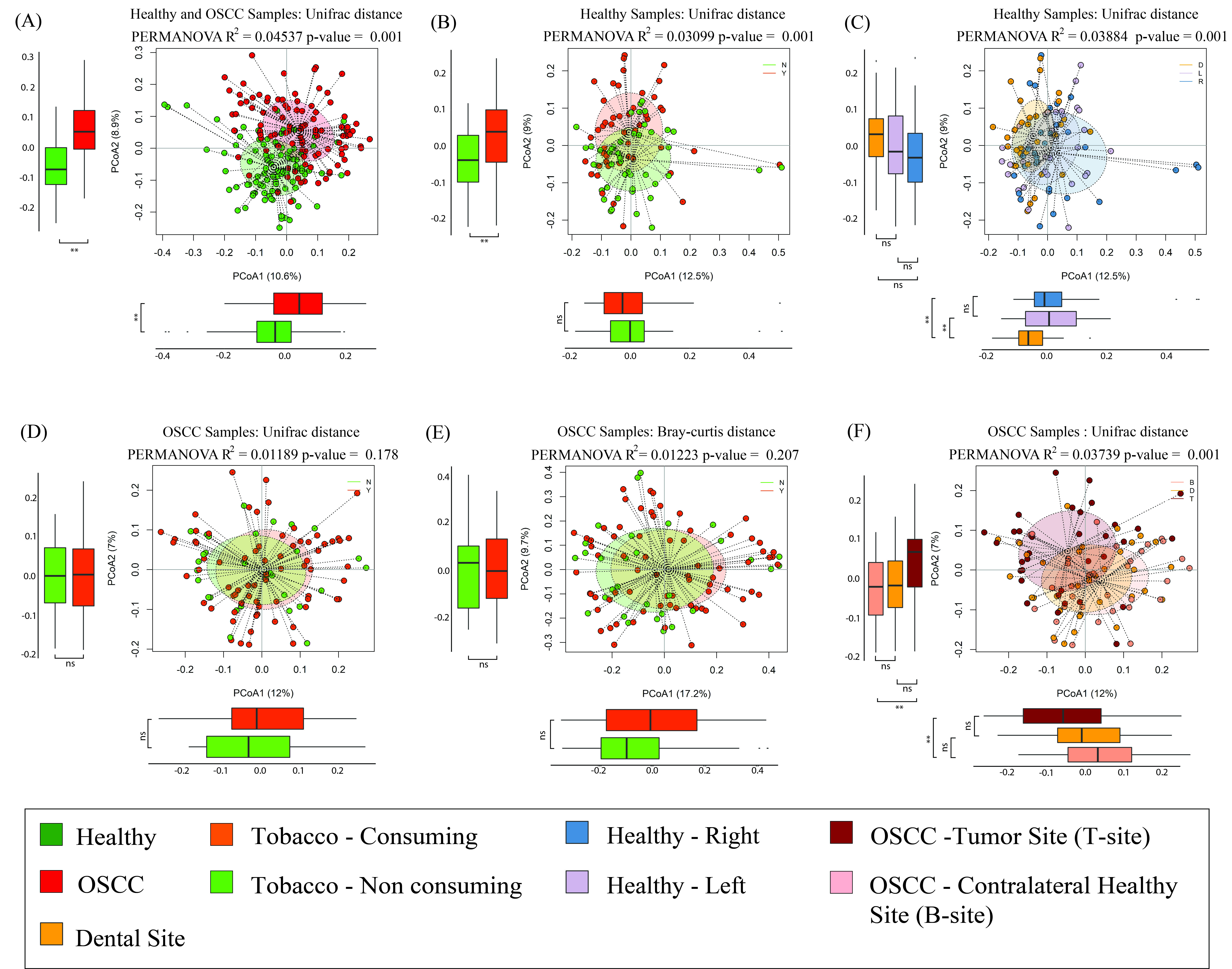

Supplement: Supplementary Figure 2 — Inter-sample diversity (beta-diversity) analysis of healthy and OSCC oral microbiome considering the smokeless tobacco consumption status and sampling sites. (A) Principal Coordinate Analysis of healthy and OSCC oral microbiome (n=196) based on inter-sample unweighted-unifrac distance. (B) Principal Coordinate Analysis of smokeless tobacco consuming and non-consuming healthy oral microbiome (n=94) based on inter-sample unweighted-unifrac distance. (C) Principal Coordinate Analysis of healthy oral microbiome (n=94) of left-, right- buccal sites and dental sites based on inter-sample unweighted-unifrac distance. (D) Principal Coordinate Analysis of smokeless tobacco consuming and non-consuming OSCC oral microbiome (n=102) based on inter-sample unweighted-unifrac distance. (E) Principal Coordinate Analysis of smokeless tobacco consuming and non-consuming OSCC oral microbiome (n=102) based on inter-sample Bray-Curtis distance. (F) Principal Coordinate Analysis of oral microbiome of tumour site, contralateral healthy site and dental site of OSCC (n=102) samples based on inter-sample unweighted-unifrac distance. [file Image_2.jpeg]

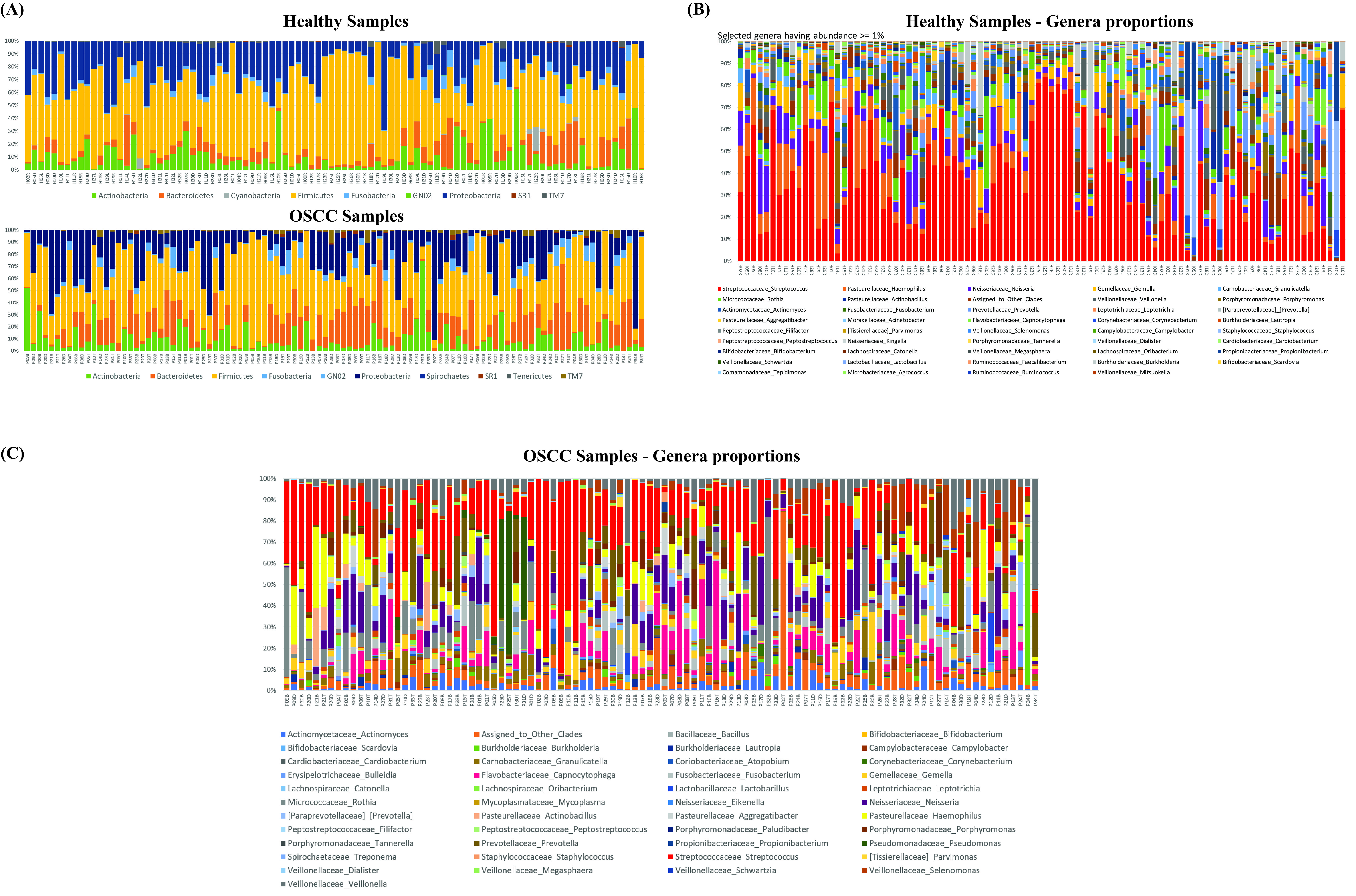

Supplement: Supplementary Figure 3 — Relative abundance of bacterial phyla and genera in healthy and OSCC oral microbiome. (A) Relative abundance of bacterial phyla in healthy and OSCC oral microbiome (n=196). (B) Relative abundance of bacterial genera in healthy oral microbiome (n=94). (C) Relative abundance of bacterial genera in OSCC oral microbiome (n=102). [file Image_3.jpeg]

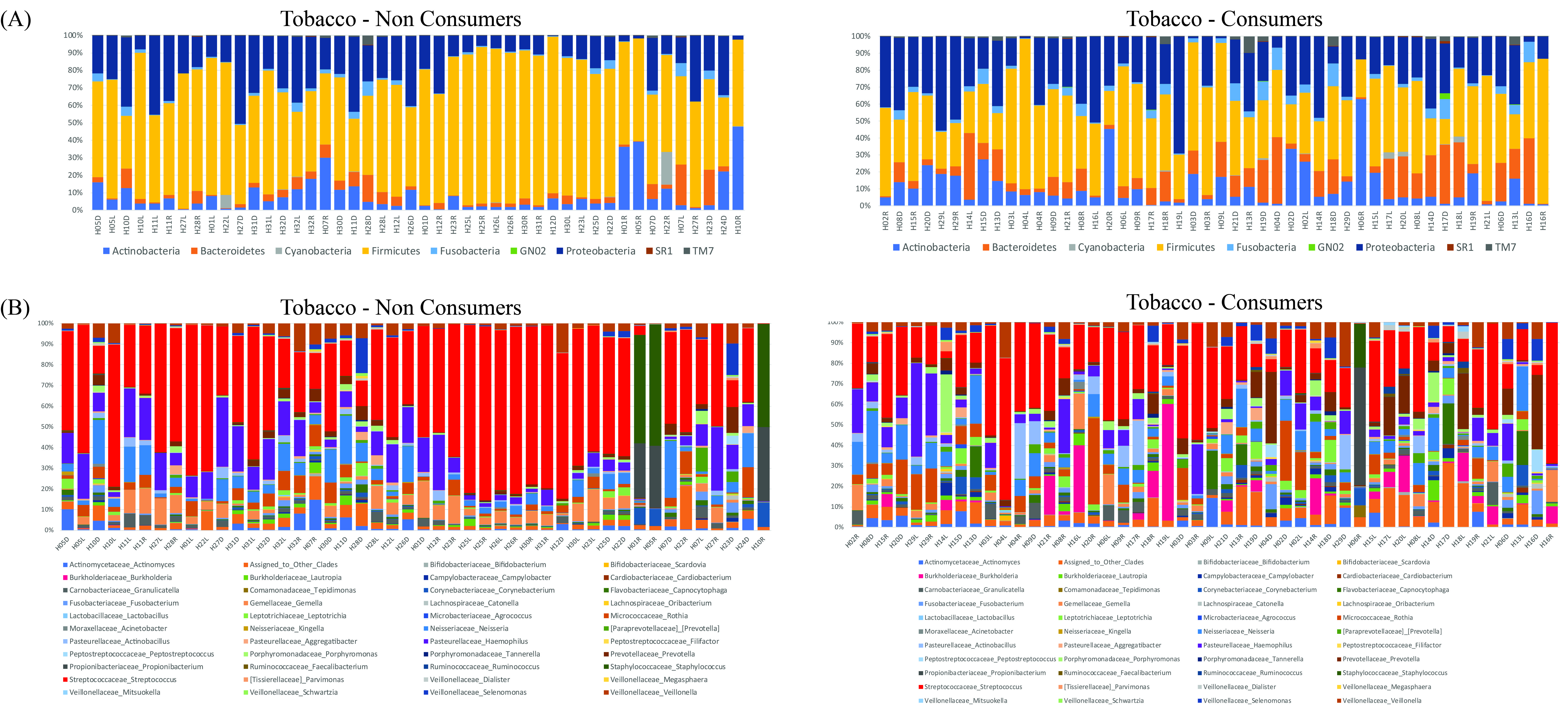

Supplement: Supplementary Figure 4 — Comparison of relative abundance of bacterial phyla and genera in smokeless tobacco consuming and non-consuming healthy samples. (A) Relative abundance of bacterial phyla in smokeless tobacco consuming and non-consuming healthy oral microbiome (n=94). (B) Relative abundance of bacterial genera in smokeless tobacco consuming and non-consuming healthy oral microbiome (n=94). [file Image_4.jpeg]

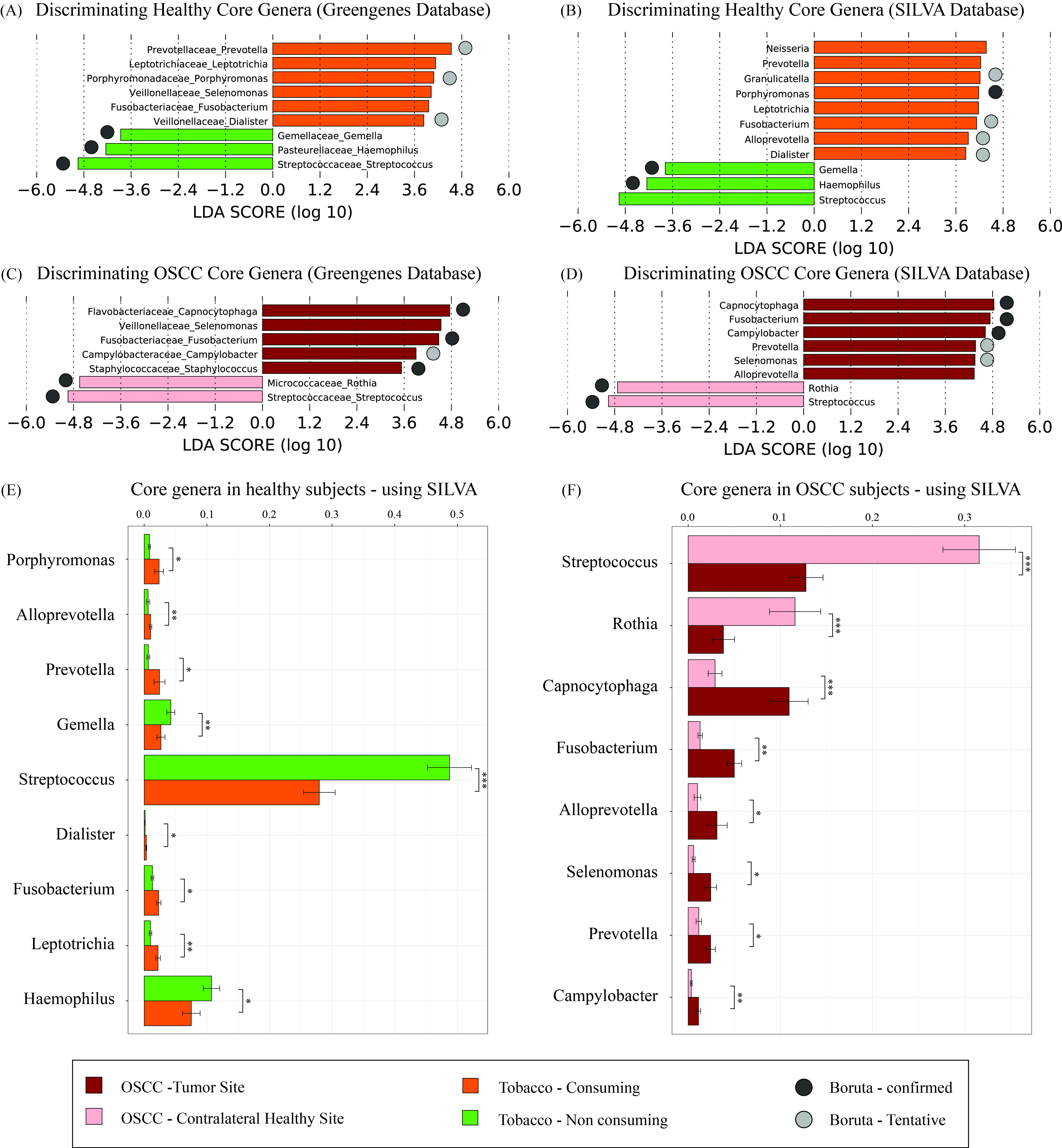

Supplement: Supplementary Figure 5 — Differentially abundant genera in healthy and OSCC samples considering smokeless tobacco consumption status and sampling sites into account. (A) Core genera that are differentially abundant in smokeless tobacco-consuming and non-consuming healthy oral microbiome. Corresponding LDA score for each genus reported by LEfSe were indicated in the figures. Taxonomic annotation of OTUs were carried out using Greengenes database. (B) Core genera that are differentially abundant in smokeless tobacco-consuming and non-consuming healthy oral microbiome. Corresponding LDA score for each genus reported by LEfSe were indicated in the figures. Taxonomic annotation of OTUs were carried out using SILVA database. (C) Core genera that are differentially abundant in tumour site and contralateral healthy buccal site of OSCC microbiome. Corresponding LDA score for each genus reported by LEfSe were indicated in the figures. Taxonomic annotation of OTUs were carried out using Greengenes database. (D) Core genera that are differentially abundant in tumour site and contralateral healthy buccal site of OSCC microbiome. Corresponding LDA score for each genus reported by LEfSe were indicated in the figures. Taxonomic annotation of OTUs were carried out using SILVA database. (E) Relative abundance of core-genera (annotated using SILVA) in smokeless tobacco consuming and non-consuming healthy samples. (F) Relative abundance of core-genera (annotated using SILVA) in tumour site and contralateral healthy site of OSCC samples. [file Image_5.jpeg]

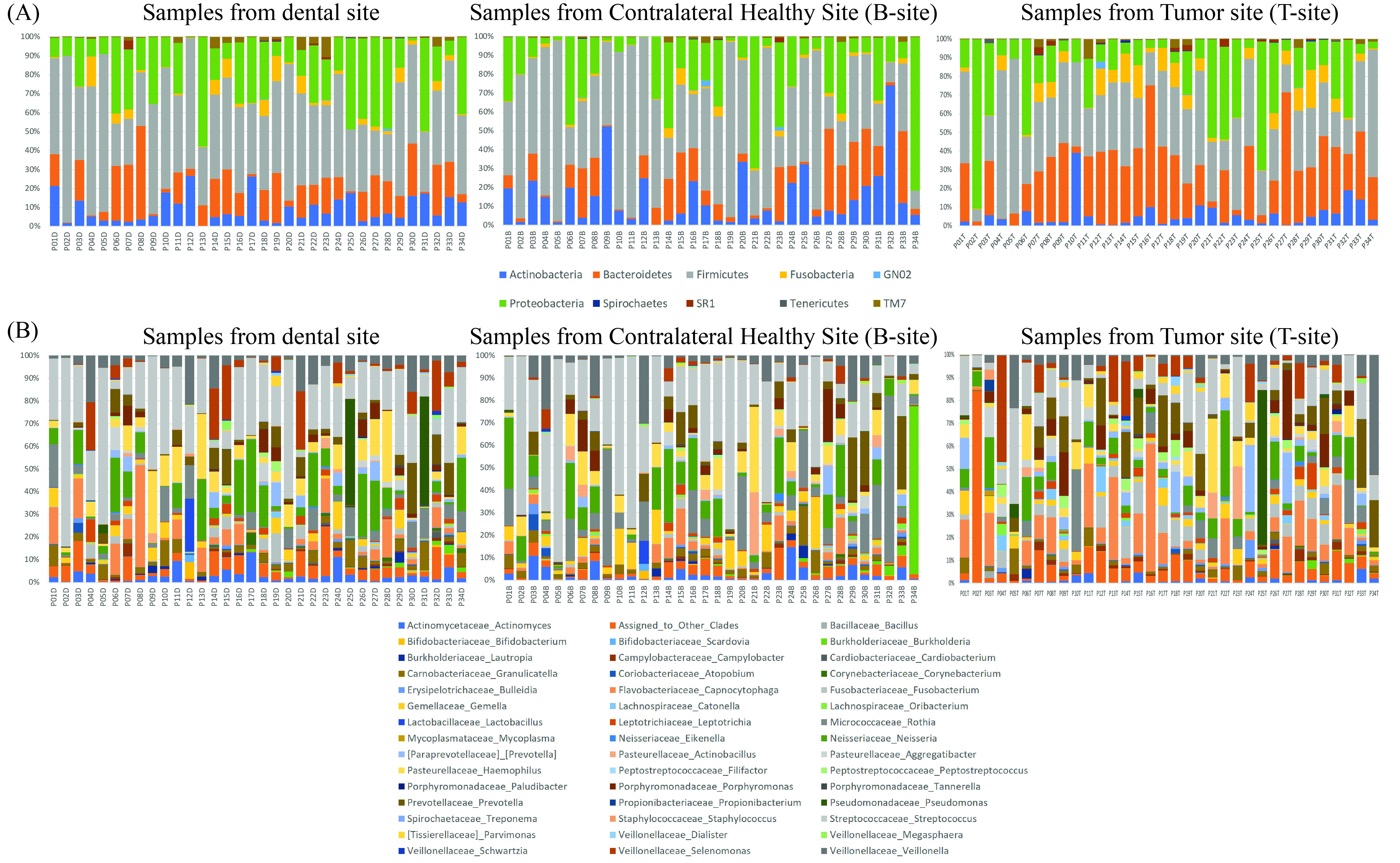

Supplement: Supplementary Figure 6 — Relative abundance of bacterial phyla and genera in OSCC microbiome. (A) Relative abundance of bacterial phyla in dental site, tumour site and contralateral healthy site. (B) Relative abundance of bacterial genera in dental site, tumour site and contralateral healthy site. [file Image_6.jpeg]

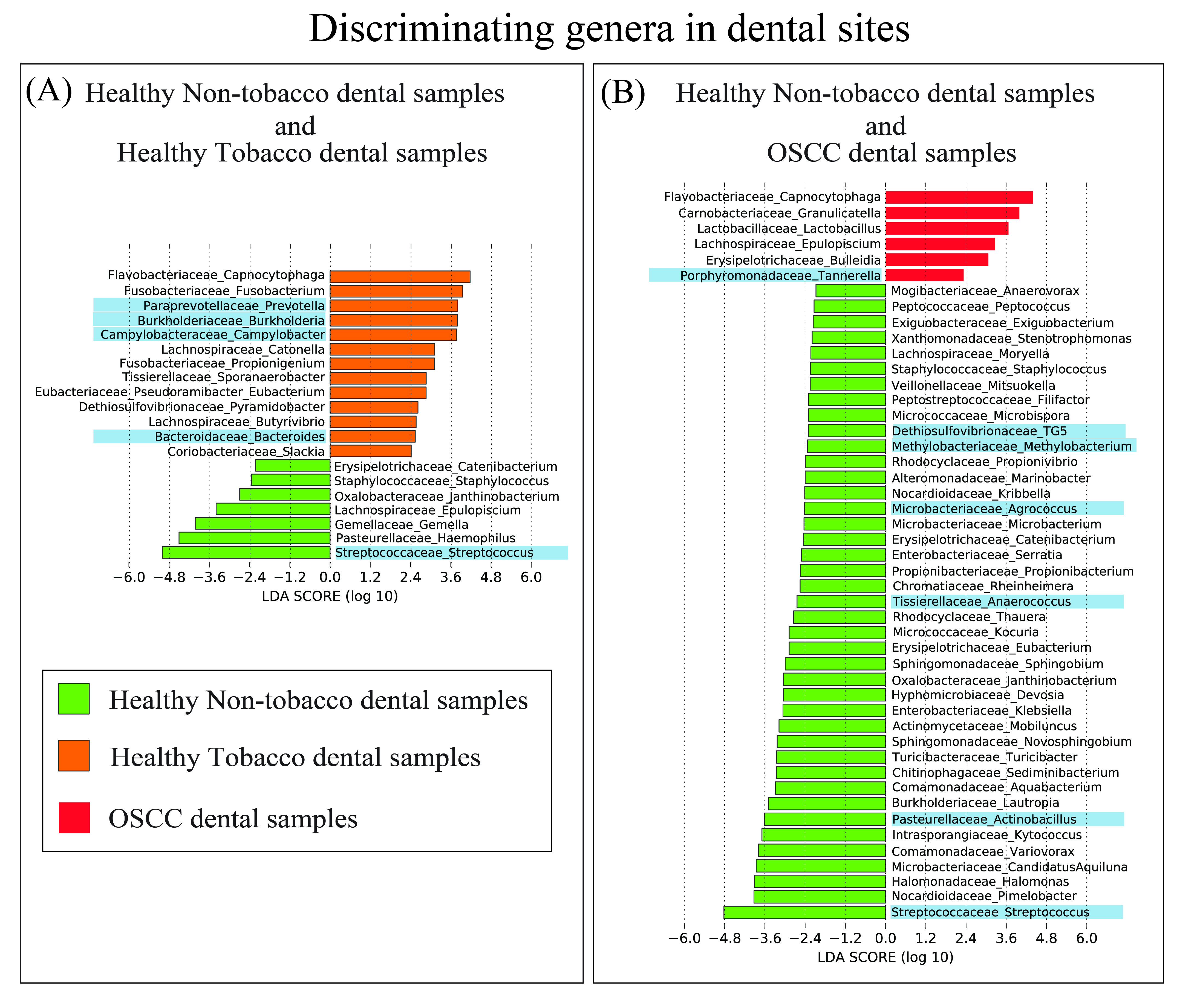

Supplement: Supplementary Figure 10 — Differentially abundant bacterial genera in dental sites of healthy and OSCC samples. (A) Genera that are differentially abundant in smokeless tobacco-consuming and non-consuming healthy dental microbiome. Corresponding LDA score for each genus reported by LEfSe were indicated in the figures. (B) Genera that are differentially abundant in smokeless tobacco non-consuming healthy dental microbiome and OSCC dental microbiome. Corresponding LDA score for each genus reported by LEfSe were indicated in the figures. [file Image_10.jpeg]

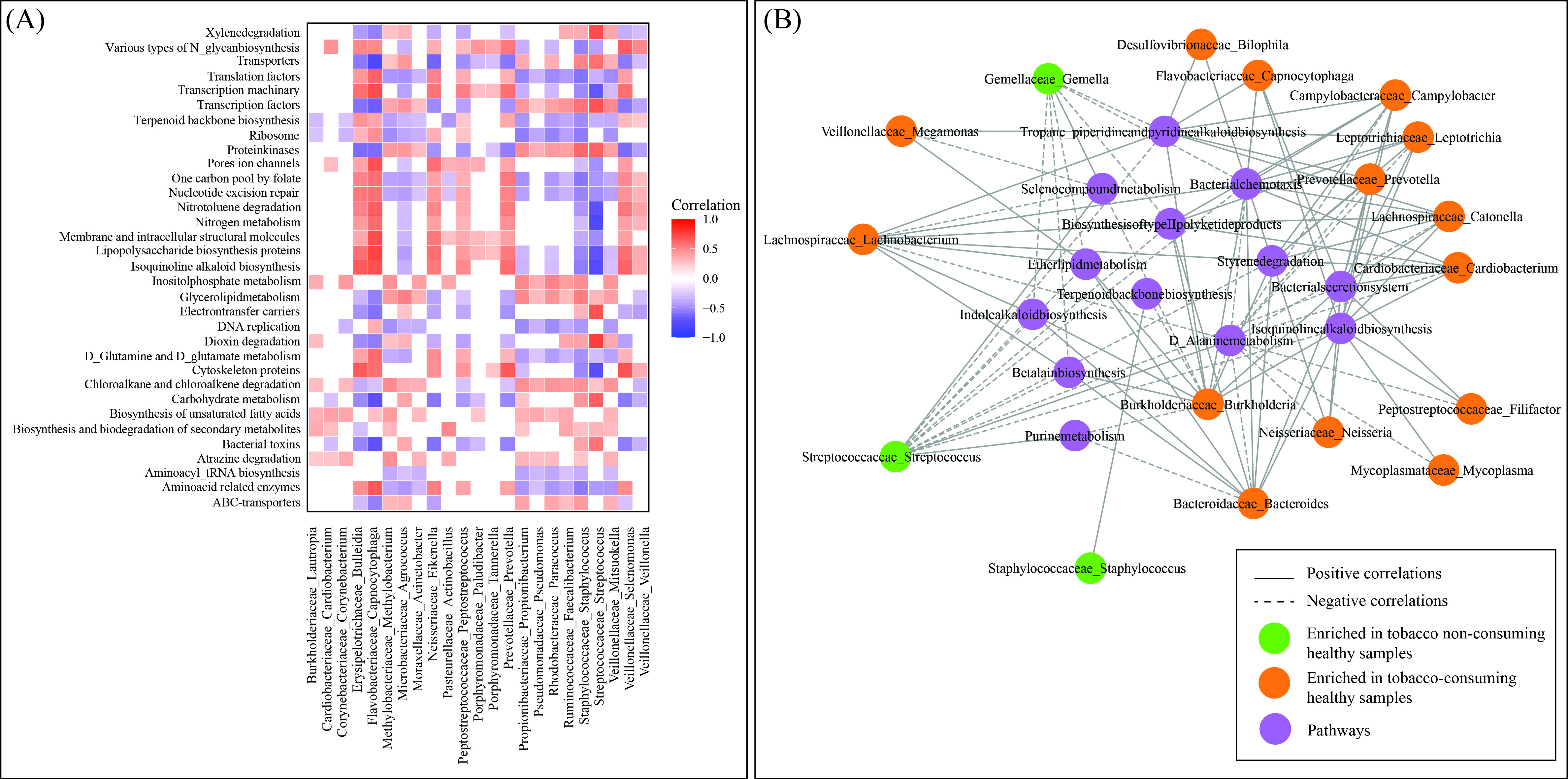

Supplement: Supplementary Figure 13 — Correlation analysis of differentially abundant genera and pathways in healthy and OSCC samples. (A) Heatmap indicating the significant correlation (Spearman) between differentially abundant genera and pathways in healthy and OSCC samples. (B) Network plot indicating the significant correlation (Spearman) between differentially abundant genera and pathways in smokeless tobacco-consuming and non-consuming healthy samples. [file Image_13.jpeg]
