# Supplementary material for: Assessing the Effect of Smokeless Tobacco Consumption on Oral Microbiome in Healthy and Oral Cancer Patients
Source: Front Cell Infect Microbiol. 2022 Mar 31;12:841465. doi: 10.3389/fcimb.2022.841465 (PMC9009303; doi:10.3389/fcimb.2022.841465)
Supplement: Supplementary file 22 [file DataSheet_1.pdf]

UPLC-MS based analysis of oral metabolome of healthy and OC samples exhibit clear separation in Principle Co-ordinate analysis (**Supplementary Figure 12A**). Sphinganine, Nedocromil sodium, Estrane, Procainamide, 1-Nitrosonaphthalene, Tolmetin, glutamine, histidine, Azelaic acid etc. were positively associated with OC samples (**Supplementary Figure 12B, C**). Since only four samples were included from healthy tobacco-consumers, metabolomic level differences due to tobacco consumption was difficult to elucidate. Dihydrosphingosine (sphinganine) is shown to be associated with OSCC samples and tobacco consuming healthy samples. Sphingolipid metabolism (e.g., sphingosine, phytosphingosine) hint at dysregulation of cell proliferation and these metabolites are potential markers were of OSCC diagnosis and evaluation of the outcome of OSCC treatment (Ogretmen, 2017; Song et al., 2020). It has been found that the content of each examined sphingolipid was markedly elevated in the cancer tissue compared with the healthy endometrium (Knapp et al., 2010). Sphingomyelin and glycosphingolipids (complex dietary sphingolipids) have been reported to inhibit the development of colon cancer (Ahn and Schroeder, 2016). This protective role may be the result of the conversion of complex sphingolipids to bioactive metabolites including sphingoid bases (sphingosine and sphinganine) and ceramide, which inhibit proliferation and stimulate apoptosis (in case of colon cancer) (Ahn and Schroeder, 2010). Both sphinganine and sphingosine at high concentrations (8–10 μM) arrested the cell cycle at G2/M 62 (in case of breast cancer) (Eun et al., 2006). Because of progressive tumour growth, host glutamine depletion develops and becomes a hallmark. Fast glutamine depletion in cancer patients is because it is essential for tumour growth and also because of cytokine-mediated alterations in glutamine metabolism in host tissues. Animal and human studies that have investigated the use of glutamine-supplemented nutrition in the host with cancer suggest that pharmacologic doses of dietary glutamine may be beneficial (Souba, 1993; Choi and Park, 2018).

References

Ahn, E. H., and Schroeder, J. J. (2010). Induction of apoptosis by sphingosine, sphinganine, and C 2-ceramide in human colon cancer cells, but not by C 2-dihydroceramide. *Anticancer Res.*

Ahn, E. H., and Schroeder, J. J. (2016). Sphingoid Bases and Ceramide Induce Apoptosis in HT-29 and HCT-116 Human Colon Cancer Cells: *https://doi.org/10.1177/153537020222700507* 227, 345–353. doi:10.1177/153537020222700507.

Choi, Y. K., and Park, K. G. (2018). Targeting glutamine metabolism for cancer treatment. *Biomol. Ther.* doi:10.4062/biomolther.2017.178.

Eun, H. A., Chang, C. C., and Schroeder, J. J. (2006). Evaluation of sphinganine and sphingosine as human breast cancer chemotherapeutic and chemopreventive agents. *Exp. Biol. Med.* doi:10.1177/153537020623101012.

Knapp, P., Baranowski, M., Knapp, M., Zabielski, P., Błachnio-Zabielska, A. U., and Górski, J.

(2010). Altered sphingolipid metabolism in human endometrial cancer. *Prostaglandins Other Lipid Mediat.* doi:10.1016/j.prostaglandins.2010.03.002.

Ogretmen, B. (2017). Sphingolipid metabolism in cancer signalling and therapy. *Nat. Rev. Cancer 2017 181* 18, 33–50. doi:10.1038/nrc.2017.96.

Song, X., Yang, X., Narayanan, R., Shankar, V., Ethiraj, S., Wang, X., et al. (2020). Oral squamous cell carcinoma diagnosed from saliva metabolic profiling. *Proc. Natl. Acad. Sci. U. S. A.* 117, 16167–16173. doi:10.1073/PNAS.2001395117/-/DCSUPPLEMENTAL.

Souba, W. W. (1993). Glutamine and cancer. *Ann. Surg.* doi:10.1097/00000658-199312000-00004.
